# Biocatalytic cascades and intercommunicated biocatalytic cascades in microcapsule systems†

**DOI:** 10.1039/d2sc01542k

**Published:** 2022-04-29

**Authors:** Pu Zhang, Amit Fischer, Yu Ouyang, Jianbang Wang, Yang Sung Sohn, Ola Karmi, Rachel Nechushtai, Itamar Willner

**Affiliations:** Institute of Chemistry, Center for Nanoscience and Nanotechnology, The Hebrew University of Jerusalem Jerusalem 91904 Israel itamar.willner@mail.huji.ac.il; Institute of Life Science, The Hebrew University of Jerusalem Jerusalem 91904 Israel; Key Laboratory of Luminescence and Real-Time Analytical Chemistry (Southwest University), Ministry of Education, College of Chemistry and Chemical Engineering, Southwest University Chongqing 400715 People's Republic of China

## Abstract

Biomolecule-loaded nucleic acid-functionalized carboxymethyl cellulose hydrogel-stabilized microcapsules (diameter *ca.* 2 μm) are introduced as cell-like containments. The microcapsules are loaded with two DNA tetrahedra, T_1_ and T_2_, functionalized with guanosine-rich G-quadruplex subunits, and/or with native enzymes (glucose oxidase, GOx, and/or β-galactosidase, β-gal). In the presence of K^+^-ions and hemin, the T_1_/T_2_ tetrahedra constituents, loaded in the microcapsules, assemble into a hemin/G-quadruplex bridged tetrahedra dimer DNAzyme catalyzing the oxidation of Amplex Red to Resorufin by generating H_2_O_2_. In the presence of co-loaded GOx or GOx/β-gal, the GOx//T_1_/T_2_ hemin/G-quadruplex cascade catalyzing the glucose-mediated oxidation of Amplex Red to Resorufin, and the three-biocatalysts cascade consisting of β-gal//GOx//hemin/G-quadruplex bridged T_1_/T_2_ catalyzing the lactose-driven oxidation of Amplex Red to Resorufin proceed in the microcapsules. Enhanced biocatalytic transformations in the microcapsules, as compared to the performance of the reactions in a homogeneous phase, are observed, due to the proximity of the biocatalysts in a confined volume. As the synthetic methodology to prepare the microcapsules yields boundaries functionalized with complementary nucleic acid tethers, the dynamic association of different microcapsules, loaded selectively with biomolecular catalysts, proceeds. The dynamic dimerization of GOx-loaded microcapsules and hemin/G-quadruplex bridged T_1_/T_2_ DNAzyme-loaded microcapsules yields effective intercommunicated microcapsules driving the GOx//hemin/G-quadruplex bridged T_1_/T_2_ DNAzyme cascade. In addition, the dynamic dimerization of GOx-loaded microcapsules with β-gal//hemin/G-quadruplex bridged T_1_/T_2_-loaded microcapsules enables the bi-directional intercommunicated operation of the lactose-stimulated three catalysts β-gal//GOx//hemin/G-quadruplex bridged T_1_/T_2_ DNAzyme cascade. The guided separation and formation of dynamic supramolecular dimer microcapsular containments, and the dictated switchable operation of intercommunicated biocatalytic cascades are demonstrated.

## Abstract 2

Dynamic dimerization of GOx-loaded microcapsules with β-gal//hemin/G-quadruplex-bridged T_1_/T_2_-loaded microcapsules guides the bi-directional intercommunication of the three catalysts cascade.

## Graphical abstract

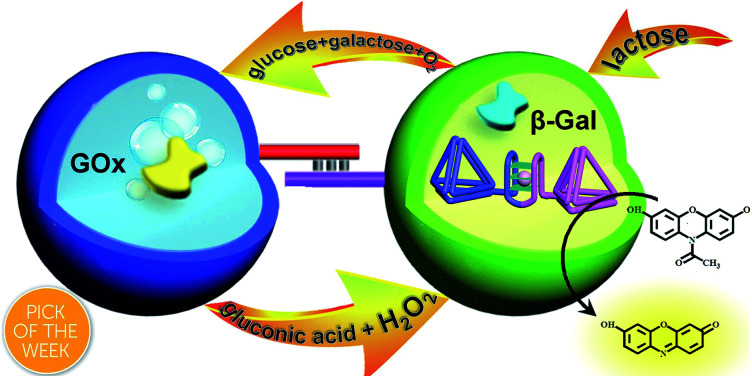

## Introduction

The construction of artificial cell-like assemblies emulating the complexity of native cells, cell-like containments, is a scientific “holy grail” attracting continuous research efforts in the area of Systems Chemistry and Systems Biology.^1^ Development of cellular containment systems addresses variety of challenging sub-goals that need to be integrated into the artificial cell-like machinery. These includes: (i) The development of cell-like containments that allow the immobilization and operation of cell-mimicking reactions and functionalities. (ii) The integration of chemically engineered ensembles into the cell-like containments capable of inducing dictated catalytic transformations (metabolism), ability to sense and response to environmental triggers, and dynamically adapt the structure, composition and chemical performance in response to external stimuli. (iii) The influx of chemical agents and the efflux of metabolites from the cell-like containments, and the guided operation of chemical transformations in the artificial containments using chemical fuels, auxiliary energy sources, such as light or redox potential, should be feasible. (iv) Appropriate chemical networks and machineries allowing motility, differentiation, exchange constituents and information transfer between the artificial cells should be integrated into the artificial containments, allowing the operation of complex catalytic cascades. Needless to state, the availability of cell-like containments combining all of these features (or more) characterizing native cells are non-existent, yet functional assemblies mimicking some of those functions were realized, and several excellent review articles summarized the advances in the field.^2^

The design of organized biocatalytic cascades on supramolecular scaffolds or biocatalytic cascades operating in confined micro- or nano-environments attracts substantial research efforts aiming to emulate bicatalytic networks in nature.^3^ The spatial proximity between the enzymes in these organized media leads to effective channeling of the product of one enzyme, acting as substrate for the neighboring enzyme, thereby, overcoming diffusional limitations and producing high local concentrations for effective intercommunication between the biocatalysts.^4^ For example, the assembly of spatially organized enzymes on DNA frameworks, such as strips,^5^ bundles^6^ or origami tiles^7^ or the organization of enzymes, on peptide ligands^8^ or protein scaffolds^9^ led to structurally ordered assemblies guiding efficient biocatalytic cascades. Similarly, the integration of multi-enzyme systems in confined environments such as microdroplets,^10^ polymersomes,^11^ or metal–organic-framework nanoparticles^12^ generated engineered containments of spatially proximate assemblies of biocatalysts for efficient operation of biocatalytic cascades. Such organized assemblies of biocatalytically active cascades were suggested as functional systems for effective sensing,^13^ bioreactors for driving efficient catalytic transformations and the synthesis of drugs.^14^

Different cell-like containments were developed including liposomes,^15^ polymersomes,^16^ proteinosomes,^17^ colloidosomes,^18^ dendrosomes,^19^ coacervate microdroplets,^20^ and were suggested as models for cells compartments. Enzymes were integrated in cell-like containments and the advantages of driving biocatalytic cascades in confined nano/micro-environments were demonstrated.^21^ Cell-like containments demonstrating motility,^22^ chemical signaling^23^ proliferation,^24^ and passive and active transport^25^ were reported. Genetic circuits^26^ and RNA catalytic networks were embedded in cell-like containments^27^ and DNA-guided communication between cell-like containments^28^ and gene expression^29^ were realized. Compartmentalization of cell-like containments and control over cascaded reactions in the compartmentalized assemblies were achieved.^30^ Also, adaptive temporal pH-responsive polymersome nanoreactor structures^31^ or transient chemical fuel-triggered microgel systems^32^ were reported, and compartmentalization of native cells was emulated by cell-like containment model systems and these revealed increased functional complexity,^33^ and control over cascaded reactions.^34^

The information encoded in the base sequence of nucleic acids provides structural and functional feature that could be utilized to design active constituents in artificial cells. For example, the base paring guided formation of duplex DNAs provides means to construct programmed duplex structures that can be dynamically interchanged by strand displacement processes controlled by the relative stabilities of the duplexes.^35^ In addition, sequence dictated dynamic reconfiguration of nucleic acid strands provides versatile means to develop switchable DNA structures, *e.g.* G-quadruplexes,^36^ triplexes^37^ or i-motif assemblies,^38^ and pre-designed nucleic acid structures, such as hairpin DNAs, or circular DNAs, introduced functional motives to operate dynamic DNA machineries, such as the hybridization chain reaction^39^ or the rolling circle amplification.^40^ Moreover, the sequence specific-nucleic acid constructs reveal selective recognition properties (aptamers)^41^ or catalytic properties (DNAzymes or nucleozymes),^42^ such as the hemin/G-quadruplex, horseradish peroxidase mimicking DNAzyme^43^ or the metal-ion-cofactor dependent DNAzymes.^44^ The nucleic acid biopolymers could substitute proteins or native enzymes, or alternatively be coupled to native enzymes, as functional constituents in artificial cells. Furthermore, the guided hybridization properties of nucleic acids led to the supramolecular assembly of 2D and 3D DNA structures, such as Y-shaped,^45^ crossover-junctions^46^ or origami frameworks.^47^ Within this context, DNA tetrahedra 3D nanostructures revealing tunable sizes and high stabilities attract substantial recent research efforts.^48^ The DNA tetrahedra nanostructures are formed upon the assembly of four appropriately sequence-engineered strands,^49^ and reveal size tunability^50^ and structural stability.^51^ By the conjugation of nucleic acid tethers to the corners of the tetrahedra or by encoding sequence specific domains into the edges of the tetrahedra structures, the functionalization of the nanostructures with nanoparticles,^52^ proteins^53^ and functional nucleic acids (DNAzymes, aptamers)^54^ were demonstrated. Various applications of DNA tetrahedra for sensing,^55^ imaging,^56^ drug carrying,^57^ and scaffolds for the organization of chiroplasmonic structures^58^ were reported. The dimensions of the DNA tetrahedra, 2.4–12.6 nm, are controlled by the length of the strands comprising the structures. As the dimensions of DNA tetrahedra are comparable to the sizes of low-molecular-weight proteins, they were suggested as structural modules that emulate proteins.^59^

In the present study, we introduce DNA-based hydrogel microcapsules as model systems for cell-like containments. The synthesis of DNA-based microcapsules and particularly the preparation of stimuli-responsive drug-loaded microcapsules has been a subject of extensive research in our laboratory.^60^ We have demonstrated the design of pH,^61^ aptamer,^62^ light^63^ and microRNA responsive^64^ microcapsules and applied the systems for programmed release of anti-cancer drugs or insulin.^65^ The study will make use of the following properties of the microcapsules to introduce the cell mimicking functions: (i) The method to prepare the microcapsules allows the programmed loading of the containment with pre-designed constituents. (ii) The methodology to synthesize the microcapsule introduces surface functionalities that allow the dynamic intercommunication between microcapsules. (iii) The hydrogel coating membrane reveals cell-like permeation and efflux properties. The resulting DNA crosslinked hydrogel coating reveals in/out permeation and efflux of low molecular weight agents, *ca.* < 5 kDa,^65^ yet higher molecular weight substance is non-permeable acrossing the membrane boundaries and reveal confined stabilities for at least three days. These properties enable the control over the catalytic transformations within the microcapsules. (iv) The introduction of catalytic DNA tetrahedra nanostructures and native enzyme into the microcapsule containments will be presented. The intracapsular DNA tetrahedra/enzyme cascades and the dynamic intercommunication of microcapsules loaded with catalytic tetrahedra/enzyme constituents and the guided operation of catalytic cascades will be introduced. (v) The advantages of dynamic intercommunication of cell-like assemblies driving biocatalytic cascades over analog biocatalytic cascades in aqueous environments are presented.

## Results and discussion

**Fig. 1:**
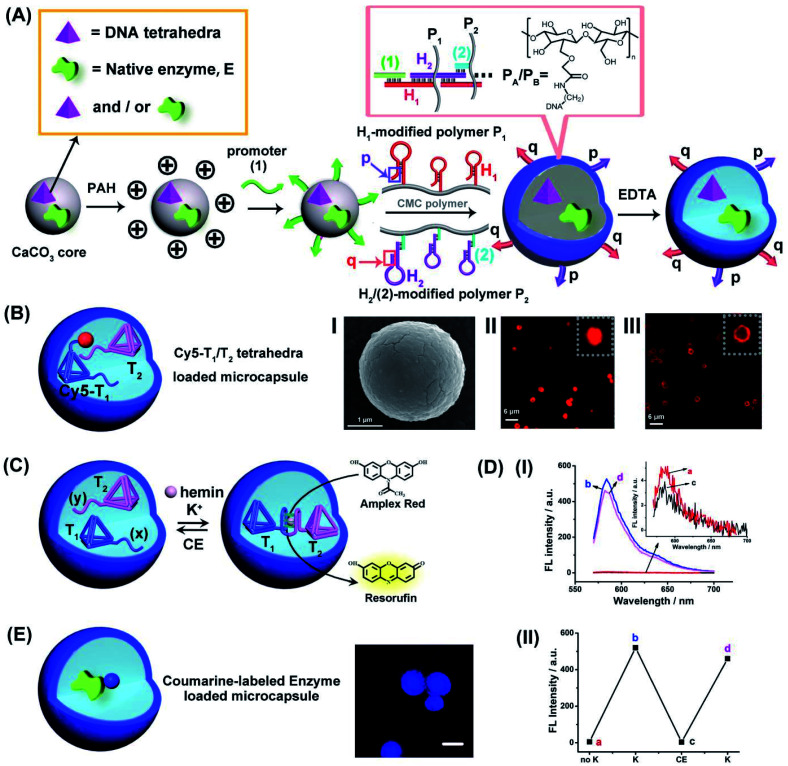
(A) Schematic preparation of DNA tetrahedra and/or native enzyme-loaded carboxymethyl cellulose (CMC) DNA duplex-crosslinked hydrogel microcapsules. (B) Schematic composition of Cy5-labeled tetrahedra T_1_/T_2_-loaded DNA duplex-crosslinked hydrogel microcapsules. Panel I-SEM image of the hydrogel-coated CaCO_3_ microparticles loaded with the Cy5-T_1_/T_2_ DNA tetrahedra. Scale bar = 1 μm. Panel II-Confocal fluorescence microscopy image of the hydrogel-coated CaCO_3_ microcapsules loaded with Cy5-labeled T_1_ and T_2_. Scale bar = 6 μm. Panel III-Confocal fluorescence microscopy image of the hydrogel-stabilized microcapsules loaded with Cy5-labeled T_1_ and T_2_ after etching of the CaCO_3_ core. Scale bar: 6 μm. (C) Cyclic K^+^-ions stimulated dimerization of the DNA tetrahedra T_1_/T_2_ by hemin/G-quadruplex DNAzyme bridges and their separation by means of 18-crown-6-ether (CE). Formation of the hemin/G-quadruplex is probed by the DNAzyme catalyzed oxidation of Amplex Red to the fluorescent Resorufin. (D) Panel I-Fluorescence spectra of the bulk solution that includes Amplex Red, 0.083 mM, hemin, 0.167 μM and H_2_O_2_, 4.16 mM upon: (a) Without K^+^-ions. (b) and (d) Cyclic addition of K^+^-ions, 50 mM. (c) Intermediate addition of 200 mM CE. Panel II-Switchable fluorescence intensities of microcapsule stimulated generation of Resorufin. (E) Schematic composition of Coumarin-labeled GOx-loaded microcapsules and confocal fluorescence microscopy image of the Coumarin-labeled GOx-loaded microcapsules. Scale bar = 2 μm.

The preparation of the loaded hydrogel microcapsules followed a synthetic path developed in our laboratory and is exemplified with the loading of a DNA tetrahedra T_1_ and/or a native enzyme, E, Fig. 1 (A). CaCO_3_ microparticles were impregnated with the DNA tetrahedra T_1_ and/or the enzyme and acted as template for the construction of the microcapsules. The T_1_/E-impregnated microparticles were coated with polyallylamine hydrochloride (PAH) and the polymer coating was modified with the promoter nucleic acid strand (1). The (1)-functionalized particles were interacted with the carboxymethyl cellulose (CMC) polymer chains P_1_ and P_2_ modified with the hairpins H_1_ and the conjugate (2)/H_2_. The hairpin H_1_ and H_2_ were pre-engineered to allow the (1)-triggered opening of H_1_ followed by the opening of hairpin H_2_ by the single strand stem domain of opened H_1_, and subsequently, opened stem domain of H_2_ hybridizes with hairpin H_1_. That is, the interaction of the (1)-modified particles with the two polymers induces the cross-opening of the hairpins H_1_/H_2_ and crosslinking of the polymer chains by the duplex H_1_/H_2_ bridges while forming a hydrogel coating on the particles. Formation of the hydrogel coating is terminated when the deposition of the polymer chains on the core particles was completed. Note, however, that the cross-opening of the hairpin constituents associated with the coating leads to surface-bound tether functionalities p and q that correspond to unused open hairpin domains of H_1_ and H_2_, respectively. (Note that hairpin H_2_ was hybridized to the strand (2) associated with P_2_ in order to follow directionality constrains for cross-opening of the two hairpins). The resulting hydrogel coated microparticles were etched with EDTA to dissolve the CaCO_3_ cores, resulting in the T_1_/E-loaded microcapsules, acting as the cell-model containment units in the present study. The resulting loaded hydrogel microcapsules reveal the following features that will be used in the present study: (i) Within the preparation procedure, the microcapsules can be loaded with mixtures of different tetrahedra, mixtures of different enzymes or mixtures of DNA tetrahedra and enzymes. (ii) As will be demonstrated, the DNA tetrahedra structures retain their structural features after the etching procedure. Also, the enzyme encapsulated in the microcapsules retain over >80% of their native activities, after the etching process. (iii) The tethers p and q associated with the hydrogel coating exhibit inherent complementary and inter-tether recognition features. As a result, the equilibrated dimerization (or oligomerization of the microcapsules is possible, or alternatively the equilibrated dimerization of microcapsules containing different loads is feasible). (iv) By appreciate labeling of the loads with fluorophores, the stability and confinement of the loads within the microcapsules can be evaluated. (v) Specifically, we apply two different tetrahedra nanostructures, T_1_ and T_2_, modified with G-quadruplex subunits (*x*) and (*y*) that self-assemble, in the presence of K^+^-ions, into G-quadruplex-bridged dimer constituents. In the presence of hemin, the association of the hemin ligand to the G-quadruplex yields the hemin/G-quadruplex DNAzyme-crosslinked tetrahedra T_1_/T_2_ dimer.

For the characterization of the loaded microcapsules, we performed a series of background experiments: (i) We loaded the microcapsules with the Cy5-labeled tetrahedra T_1_ and non-labeled T_2_, Fig. 1(B), and probed the confinement of the tetrahedra to microcapsule containment. Fig. 1(B), panel I, depicts the SEM image of the Cy5-labeled tetrahedra T_1_-loaded microparticles, and the confocal fluorescence microscopy images of the resulting hydrogel-coated Cy5-modified T_1_ loaded microparticles before etching, panel II, and of the Cy5-modified T_1_-loaded microcapsules after etching, panel III. Microcapsules revealing an average diameter corresponding to 2–3 μm are formed. (ii) To validate the intact structure of the tetrahedra loads in microcapsular containment after the synthesis and etching of the core CaCO_3_ microcapsules, we performed a control experiment where the CaCO_3_ particle were impregnated with the T_1_-tetrahedra and subsequently etched with EDTA, at similar condition used for the preparation of the microcapsules. The resulting solution containing the tetrahedra was analyzed by electrophoresis and compared to a standard tetrahedra solution. The result and accompanying discussion are presented in Fig. S1.† Pure, intact tetrahedra structure were observed suggesting that the subsequent etching of the core CaCO_3_ have no perturbing effect on the tetrahedra nanostructures. (For further characterization of the tetrahedra structures by electrophoretic separation, see ESI, Fig. S2,† and the characterization of the monomer tetrahedra or dimer tetrahedra nanostructures by atomic force microscopy (AFM) imaging, see ESI, Fig. S3†). (iii) The resulting tetrahedra-loaded microcapsules are stable for at least three days, Fig. S4.† No leakage of the tetrahedra from the microcapsule containments could be detected within this time-interval. (iv) The loading of the tetrahedra T_1_ and T_2_ in the microcapsules was evaluated to be 0.1 μM and 0.09 μM, respectively, by separate labeling T_1_ with Cy5 and T_2_ with Cy3 in the loaded mixture of T_1_/T_2_ and by using appropriate calibration curves of Cy5-labeled T_1_ and Cy3-labeled T_2_ (For details, see ESI Fig. S5†). (v) The two tetrahedra structures, T_1_/T_2_, were loaded in the microcapsular containments, treated with K^+^-ions, in presence of hemin as cofactor, to probe the formation of the hemin/G-quadruplex crosslinked tetrahedra dimer structure and its catalytic activity to stimulate the catalyzed oxidation of Amplex Red to the fluorescent Resorufin product, Fig. 1(C). The results in Fig. 1(D), panel I demonstrate the permeation of K^+^-ions and hemin into the T_1_/T_2_ loaded microcapsules, and the formation of the hemin/G-quadruplex DNAzyme in the presence of Amplex Red and H_2_O_2_, results in the activation of the hemin/G-quadruplex DNAzyme that catalyzes the oxidation of Amplex Red to Resorufin. Furthermore, Fig. 1(D), panel II, demonstrates that upon the subsequent permeation of 18-crown-6-ether, CE, into the microcapsule containment, the hemin/G-quadruplex can be separated and its catalytic activity is switched off, and by the cyclic permeation of K^+^-ions/CE into the microcapsules, the DNAzyme activity can be reversibly switched between “ON” and “OFF” states. (For further characterization of the switchable formation and dissociation of the G-quadruplex-bridged tetrahedra dimer, using a fluorescent Zn(ii)-protoporphyrin IX, Zn(ii)-PPIX, fluorescent label, see Fig. S6–S8,† and accompanying discussion). (vi) In addition, we applied the procedure outline in Fig. 1(A) to load different enzymes into the microcapsule containments. (*e.g.*, glucose oxidase, GOx, and/or β-galactosidase, β-gal). By labeling of the enzymes with appropriate fluorophores, the fluorophore-functionalized enzyme-loaded microcapsules allow the fluorescence imaging of the resulting biocatalytic capsules. For example, Fig. 1(E) depicts the resulting coumarin-modified glucose oxidase (GOx)-loaded microparticles and the confocal fluorescence microscopy image of the microcapsules. Using an appropriate calibration curve relating the fluorescence intensities to the concentration of the modified enzyme and knowing the number of microcapsules, the loading of the labeled enzyme in the microcapsules is evaluated. (For the specific system, a loading corresponding to 0.026 nM coumarin-labeled GOx per microcapsule was evaluated). Furthermore, by the integration of a non-modified enzyme in the microcapsules, and assuming a similar loading degree to that of the labeled enzymes, and the activity of the enzyme entrapped in the microcapsules was followed, thus allowing the evaluation of the respective enzyme activity after the preparation and etching of the microcapsules. Along the study, mixtures of the catalytic DNA tetrahedra and the respective enzymes will be integrated in the microcapsular containments to stimulate catalytic cascade and intercommunicated catalytic cascades. The methods described above will be used to characterize different assemblies.

**Fig. 2:**
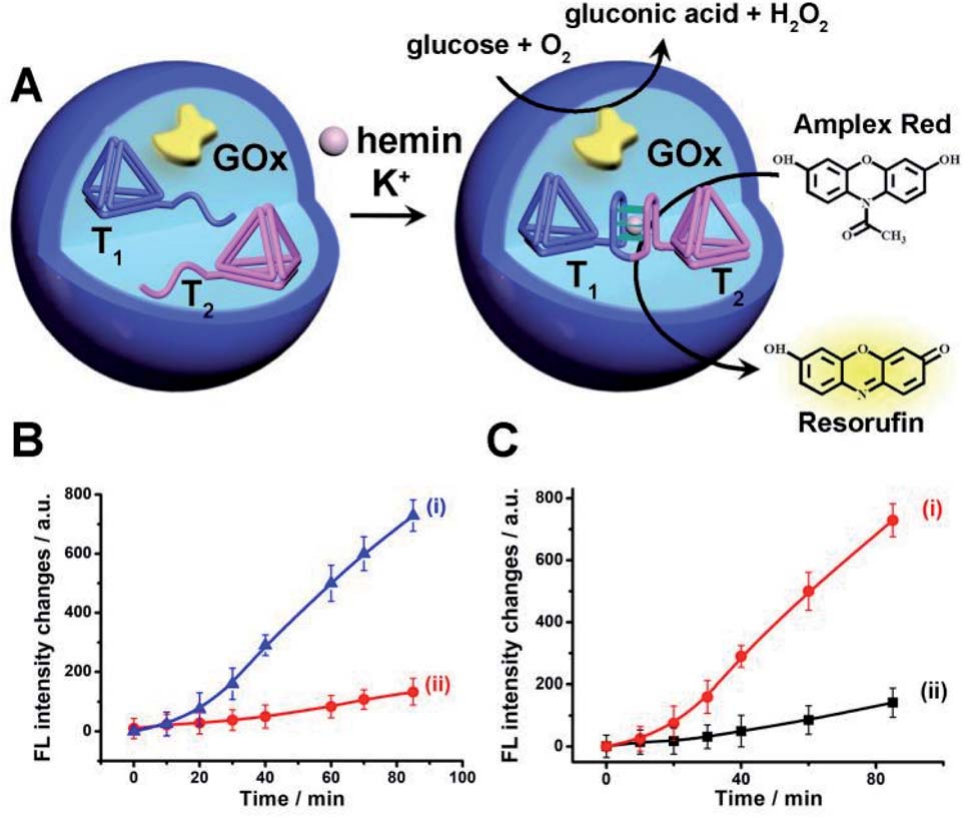
(A) Schematic operation of a catalytic cascade consisting of GOx and hemin/G-quadruplex bridged tetrahedra dimer T_1_/T_2_ loaded in a microcapsule. The GOx catalyzed aerobic oxidation of glucose yields H_2_O_2_ that drives the oxidation of Amplex Red to the fluorescent Resorufin product. (B) Time-dependent fluorescence changes of Resorufin generated by: (i) The microcapsules loaded with GOx and the separated tetrahedra, T_1_ and T_2_, in the presence of glucose, 10 mM, Amplex Red, 0.083 mM, hemin, 0.167 μM, in the presence of K^+^-ions, 50 mM. (ii) The reaction mixture described in (i) in the absence of K^+^-ions. (C) Time-dependent fluorescence intensities of Resorufin generated by: (i) The GOx//hemin/G-quadruplex bridged tetrahedra T_1_/T_2_ confined to the microcapsules. (ii) The GOx//hemin/G-quadruplex constituents in a homogeneous phase at the same concentrations of the catalysts present in the microcapsules. GOx, 0.063 μM, hemin/G-quadruplex bridged tetrahedra T_1_/T_2_ dimer 0.07 μM, glucose, 10 mM, Amplex Red, 0.083 mM, hemin, 0.167 μM and K^+^-ions 50 mM. Error bars derived from *N* = 3 experiments.

The first system to be addressed is displayed in Fig. 2. The two tetrahedra T_1_/T_2_ and GOx were loaded in the microcapsules. The loading of T_1_/T_2_ and GOx in the microcapsules were evaluated by the alternate loading of the capsules with mixtures that include the fluorophore Cy5-labeled T_1_, Cy5-labeled T_2_, and coumarine-labeled GOx to be 0.063 μM, 0.07 μM and 0.12 μM, respectively. (The calibration curve of coumarin-labeled GOx is shown in Fig. S9†). Treatment of the T_1_/T_2_ and GOx-loaded microcapsules with K^+^-ions and hemin resulted in the formation of the hemin/G-quadruplex bridged T_1_/T_2_ DNAzyme in the capsules. The subsequent treatment of the capsules with glucose and Amplex Red resulted in the intracapsular operation of the GOx//hemin/G-quadruplex cascade where GOx catalyzed the aerobic oxidation of glucose to gluconic acid and H_2_O_2_, and the resulting H_2_O_2_ mediated the hemin/G-quadruplex catalyzed oxidation of Amplex Red to the fluorescent product Resorufin. The biocatalytic cascade was probed by following the time-dependent formation of Resorufin in the microcapsules, Fig. 2(B), curve (i). Fig. 2(B), curve (ii), shows the time-dependent fluorescence intensities of the same system upon excluding K^+^-ions. No fluorescence changes are observed indicating that the K^+^-ions stabilized formation of the hemin/G-quadruplex DNAzyme is essential for operating the DNAzyme cascade in the microcapsules. Fig. S10† shows the fluorescence spectra of Resorufin at different time-intervals upon treatment of the cell-like microcapsules with different concentrations of glucose. As the concentration of glucose increases, the biocatalytic cascade is intensified. In addition, the progress of the biocatalytic cascade in the microcapsular heterogeneous system was compared to the operation of the bicatalysts hemin/G-quadruplex bridged T_1_/T_2_ and GOx in a homogeneous buffer solution that included the catalysts at bulk concentrations identical to the concentrations of the catalysts in the microcapsules. (All other constituents, *e.g.* K^+^-ions, hemin, Amplex Red and glucose were identical to those present in the microcapsular system). Fig. 2(C), curve (ii), presents the time-dependent formation of Resorufin in the homogeneous buffer solution, in comparison to the formation of Resorufin in the microcapsular system, curve (i). The biocatalytic cascade in the microcapsular system is 6-fold enhanced as compared to the homogeneous system. This is attributed to generation of the bicatalyst cascade in the confined volume of the microcapsules. That is, the GOx-biocatalyzed formation of H_2_O_2_ in spatial proximity to the hemin/G-quadruplex DNAzyme that leads to the effective oxidation of Amplex Red to Resorufin as a result of effective channeling of H_2_O_2_ to the hemin/G-quadruplex DNAzyme. Similar rate enhancement of bicatalysts cascades in confined microenvironments were previously demonstrated,^3^ for example, by the spatial proximate organization of two enzymes on DNA templates,^5,6^ DNA origami structures,^7^ microdroplets^10^ or other cell-like containments.^11^ The confinement effect of the microcapsules on enhancing biocatalytic cascades was further demonstrated in a three biocatalyst-loaded microcapsule containment assembly. In this system, the hemin/G-quadruplex bridged T_1_/T_2_ horseradish peroxidase mimicking DNAzyme was coupled to GOx and β-galactosidase, β-gal, using lactose as the parent substrate. In this system, Fig. S11† (A), β-gal hydrolyses lactose to glucose and galactose, the resulting glucose is aerobically oxidized gluconic acid and H_2_O_2_, and the resulting H_2_O_2_ acts as intracapsular oxidant for the hemin/G-quadruplex bridged T_1_/T_2_ DNAzyme-catalyzed oxidation of Amplex Red to Resorufin. The characterization of this three biocatalysts-loaded microcapsule system and the results corresponding to the operation of the three catalysts are presented and discussed in detail in Fig. S12–S15.† As for the two-catalysts-loaded microcapsule system, we find that the three biocatalysts-loaded microcapsules reveal a 5-fold rate enhancement, as compared to the three catalysts cascade in a homogeneous buffer phase, due to the operation of the cascaded catalysis in the confined microenvironment of the microcapsule.

**Fig. 3:**
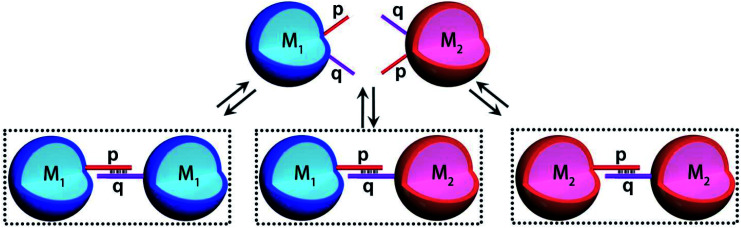
Schematic dynamic dimerization paths of the different types of microcapsules, M_1_ and M_2_ loaded with Coumarin-labeled GOx (M_1_) and Cy5-labeled T_1_ and unlabeled T_2_ (M_2_) using surface-linked nucleic acid tethers (p and q) residues of the HCR hairpins H_1_/H_2_ used to generate the microcapsules.

The introduction of DNA-based hydrogel microcapsules as cell-like containments, allows, however, the use of the microcapsules as functional units to establish intercommunication between the microcapsules, while the discussion till now addressed the loading and catalytic cascade in the microcapsule containments, the method fabricating the microcapsules provides structural features that eventually allow the design of intercommunicating microcapsules. The cross-opening hairpins H_1_ and H_2_ associated with the polymer chains P_1_ and P_2_ leads to a duplex-bridged hydrogel boundary that stabilizes the microcapsules. The synthesized hydrogel matrices yield, however, microcapsules that contain non-hybridized free, self-complementary tethers of H_1_ and H_2_ at the microcapsule membrane boundaries. Albeit, the surface coverage of these tethers is low, the tethers could provide important means to intercommunicate between microcapsules that include different loads. This is schematically outlined in Fig. 3, where two hydrogel microcapsules M_1_ and M_2_ potentially, loaded with different loads are interacted. The tethers p, q, exhibiting half complementarity represent segment of the H_1_ and H_2_ opened hairpin tethers, and thus dynamic supramolecular dimerization (or eventually enhanced oligomerization) may proceed to yield M_1_ and M_2_ monomers and M_1_ and M_2_ microcapsular dimers. The spatial proximity between the microcapsules M_1_/M_2_, and the permeability of the microcapsular boundaries to low-molecular-weight agents might allow effective transport across the boundaries from microcapsules M_1_ and M_2_ or from M_2_ to M_1_ without diffusion to the bulk medium. Such mechanism could, then, provide the basis for the intercommunication biocatalytic processes occurring in neighboring microcapsules.

**Fig. 4:**
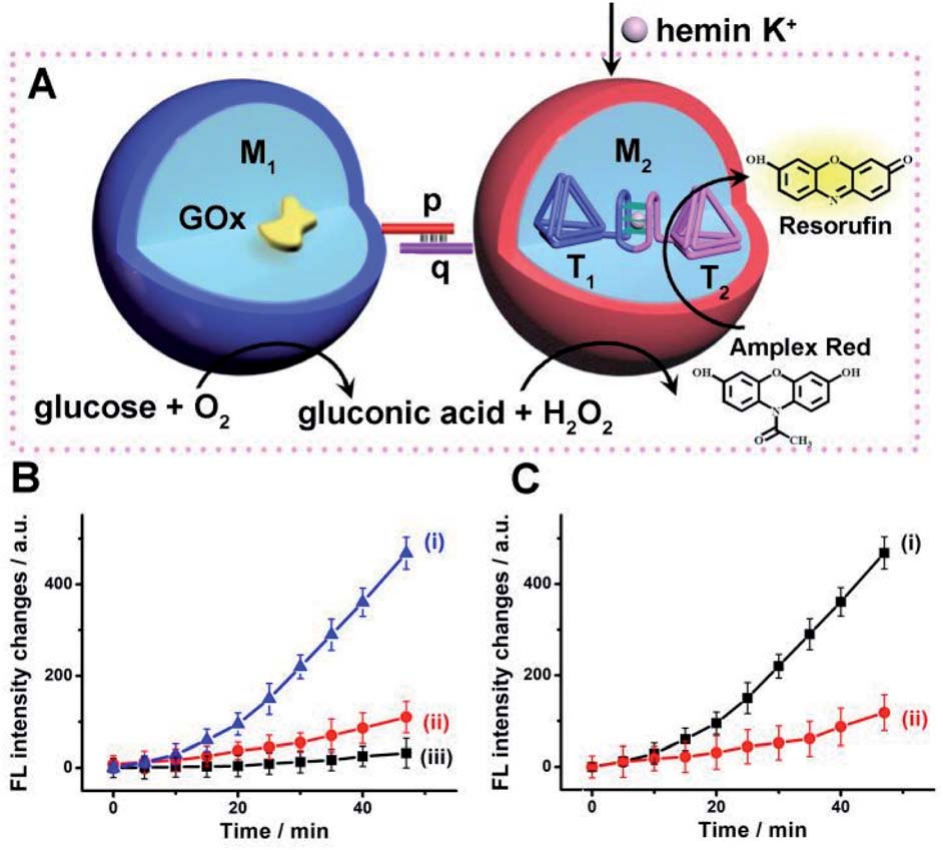
(A) Schematic intercommunication between two types of loaded microcapsules: M_1_-microcapsule loaded with GOx; M_2_-microcapsule loaded with the hemin/G-quadruplex bridged T_1_/T_2_ dimer. Intercommunication leads to the activation of the biocatalytic cascade. (B) Time-dependent fluorescence changes of Resorufin generated by: (i) The intercommunicated M_1_/M_2_ microcapsules consisting of GOx and hemin/G-quadruplex bridged T_1_/T_2_ loads. Microcapsules, in the presence of K^+^-ions, 50 mM. (ii) The intercommunicated M_1_/M_2_ microcapsules in the absence of K^+^-ions. (iii) Upon addition of CE to the K^+^-ions containing M_1_/M_2_ microcapsule mixture. In all experiments, hemin, 0.167 μM, glucose, 30 mM, and Amplex Red, 0.083 mM, are added to the system. (C) Time-dependent fluorescence changes of Resorufin generated by: (i) The intercommunicated M_1_/M_2_ microcapsules loaded with GOx, 0.12 μM, and the hemin/G-quadruplex bridged T_1_/T_2_ dimer, 0.1 μM, in the presence of K^+^-ions, 50 mM. (ii) The homogeneous mixture of GOx and hemin/G-quadruplex-bridged tetrahedra T_1_/T_2_ at concentration identical to those in the M_1_/M_2_ microcapsules. All experiments included K^+^-ions, 50 mM, hemin, 0.167 μM, glucose, 30 mM, and Amplex Red, 0.083 mM. Error bars derived from *N* = 3 experiments.

Accordingly, a mixture of two microcapsules M_1_, loaded with GOx (loading content of 0.12 μM), and microcapsules M_2_ that were loaded with the K^+^-ions-stabilized hemin/G-quadruplex-bridged tetrahedra T_1_/T_2_ (loading content of 0.1 μM) was subjected to glucose, with the attempt to drive the intercommunicated biocatalytic cascade in the presence of Amplex Red, Fig. 4(A). The inter-microcapsule biocatalytic cascade proceeds effectively, Fig. 4(B), curve (i), where the aerobic oxidation of glucose in microcapsule M_1_ yields H_2_O_2_ that diffuses through the microcapsules into the spatially proximate microcapsule M_2_, where the hemin/G-quadruplex catalyzed oxidation of Amplex Red to the fluorescent Resorufin proceeds. The effectiveness of the inter-microcapsule cascade is controlled by presence of K^+^-ions. Fig. 4(B), curve (ii), shows the cascaded catalysis of the intercommunicated M_1_/M_2_ microcapsules in the absence of K^+^-ions. Upon addition of CE to the K^+^-ions containing M_1_/M_2_ microcapsule mixture, the cascaded catalysis was inhibited due to the separation of the hemin/G-quadruplex-bridged tetrahedra T_1_/T_2_, Fig. 4(B), curve (iii). The effectiveness of the inter-microcapsule cascade is, also, controlled by the concentration of glucose, Fig. S16† shows the fluorescence spectra of Resorufin at different time-intervals upon treatment the microcapsules with different concentrations of glucose. Control experiments revealed that no leakage of the microcapsules constituents into the bulk solution occurred, as evidenced by precipitation of the microcapsules mixture (after three days) and subjecting the solution to K^+^-ions, glucose, hemin and Amplex Red (Fig. S17,† curve (i)). No Resorufin could be detected in the bulk solution, implying that no leakage of the constituents from the microcapsules occurred (Fig. S17,† curve (ii)). Therefore, the loaded microcapsules M_1_, M_2_ mixture retained their intact compositions and activities after a time-interval of three days. For further experiments demonstrating that the biocatalytic cascade originates from intimate contact between the microcapsules M_1_/M_2_, *vide infra*. Fig. 4(C) curve (ii) depicts the rate of oxidation of Amplex Red to Resorufin by a homogeneous mixture of GOx and the hemin/G-quadruplex tetrahedra, at similar concentrations present in the microcapsular microcapsules, upon subjecting to the biocatalysts glucose 30 mM, as compared to the biocatalytic cascade in the microcapsular system, Fig. 4(C), curve (i). Evidently, the biocatalytic cascade driven by the inter-communicating microcapsules is 5-fold enhanced as compared to homogeneous mixture of biocatalytic constituents.

**Fig. 5:**
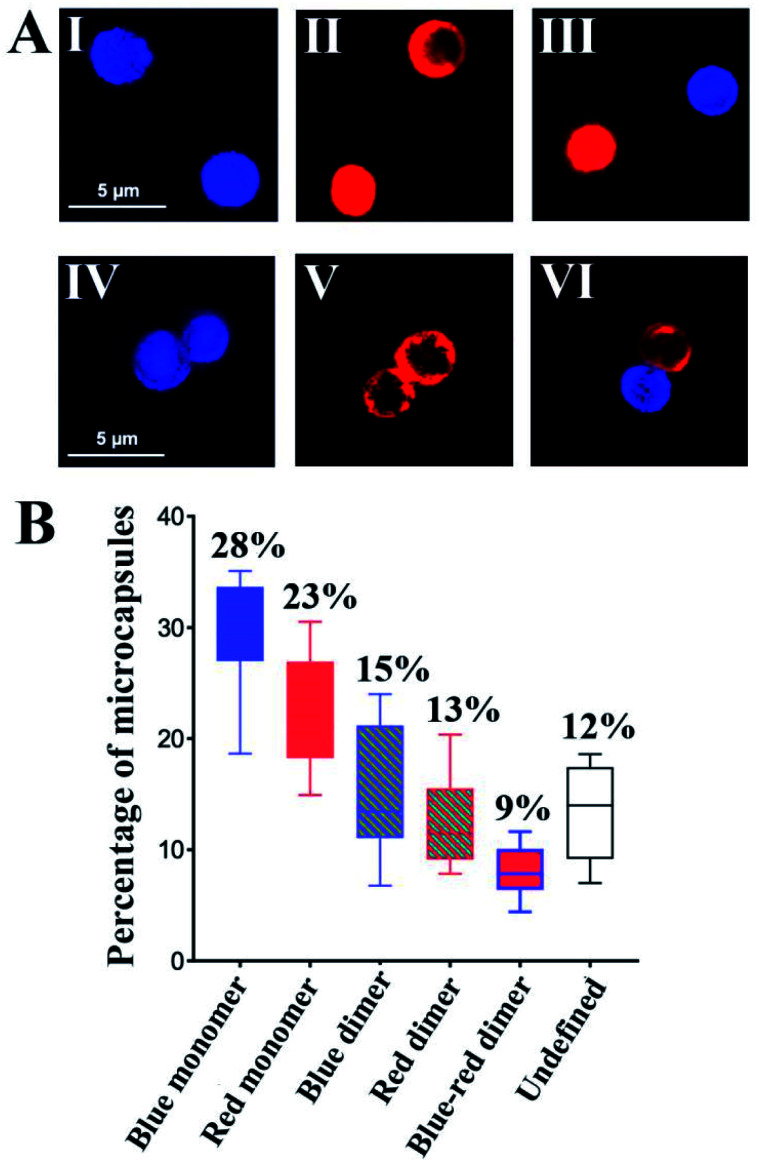
(A) Examples of confocal microscopy images corresponding to: upper row-monomer microcapsules. Panel I-Coumarin-labeled GOx-loaded microcapsules (blue fluorescence). Panel II-Cy5-T_1_/T_2_-loaded microcapsules (red fluorescence). Panel III-Separated coumarin-labeled GOx-loaded microcapsules (blue fluorescence) and Cy5-T_1_/T_2_-loaded microcapsules (red fluorescence). Bottom row-dimer microcapsules. Panel IV-Coumarin-labeled GOx-loaded microcapsules (blue fluorescence). Panel V-Cy5-T_1_/T_2_-loaded microcapsules (red fluorescence). Panel VI-Interlinked Coumarin-labeled GOx-loaded microcapsules and Cy5-T_1_/T_2_-loaded microcapsules (blue-red fluorescence). (B) Statistical analysis of confocal fluorescence microscopy fields of the population of different monomer/dimer microcapsules assemblies, in a graphic form consisting of boxes and whiskers as erroer bar types, (total 30 fields). (For blue fluorescence: *E*_x_ = 405 nm, *E*_m_ = 430–470 nm. For red fluorescence: *E*_x_ = 640 nm, *E*_m_ = 650–700 nm). Error bars derived from *N* = 3 experiments.

To further support the spatial intercommunication of the microcapsules through the dynamic interlinking of the microcapsules, a set of complementary experiments was performed. A mixture of coumarin-labeled GOx loaded microcapsules, 20 μL (*ca.* 4500 microcapsules per μL) and microcapsules loaded with Cy5-labeled T_1_ and T_2_, 20 μL (*ca.* 4500 microcapsules per μL) were allowed to equilibrate. The mixture was subjected to confocal fluorescence imaging to probe the presence of monomer microcapsules loaded with coumarin-labeled GOx (blue fluorescence), monomer Cy5-T_1_/T_2_-loaded microcapsules (red fluorescence), and dimer microcapsules consisting of dimer coumarin-labeled GOx microcapsules (blue–blue), dimer Cy5-T_1_/T_2_-loaded microcapsules (red–red) and mixed coumarin-loaded microcapsules and Cy5-T_1_/T_2_-loaded microcapsules (blue–red). Fig. 5(A) exemplifies images of the respective microcapsules. By analyzing ten different fluorescent domains of three different mixture samples (total 30 field frames), the respective monomer and dimer constituent were counted, and the average contents of the respective constituents were evaluated and presented in Fig. 5(B). The mixture includes *ca.* 51% monomer microcapsules and 37% dimer microcapsules and 12% of non-defined structures. The importance of this experiment is the demonstration of monomer and dimer microcapsules and the quantitative evaluation of the different constituents in the microcapsules mixture. Analog experiments were performed on the respective coumarin-labeled GOx-loaded microcapsules only, and on the Cy5-T_1_/T_2_-loaded microcapsules only, and these are presented in the ESI† (For quantitative evaluation of the monomer/dimer mixture, see Fig. S18 and S19† and accompanying discussion).

To further support the equilibrated monomers/dimers composition in the mixture, fluorescence-activated cell sorting (FACS) experiments were performed, Fig. S20,† and accompanying discussion. These experiments indicated *ca.* 35% of Cy5-T_1_/T_2_-loaded microcapsules including monomers and dimers, 35% of coumarin-labeled GOx loaded microcapsules containing monomer and dimer, and *ca.* 7% population of the dimer structure of the coumarin-labeled GOx loaded microcapsules and Cy5-T_1_/T_2_-loaded microcapsules in microcapsular monomer/dimer mixture, which is in good agreement with the confocal fluorescence microscopy analysis.

**Fig. 6:**
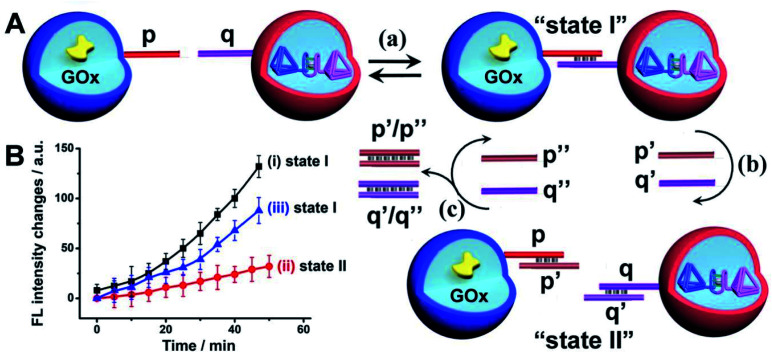
(A) Switchable inhibition of the intercommunicated microcapsule GOx//hemin/G-quadruplex biocatalyst cascade using a “fuel” inhibiting strands p′, q′ and “counter-fuel strands” p′′, q′′. (B) Time-dependent fluorescence changes corresponding to Resorufin generated by the intercommunicated GOx//hemin/G-quadruplex bridged T_1_/T_2_ cascade as a result of (a)-equilibrated p/q interlinked microcapsule dimers, state I, curve (i). (b) Upon separation of the dimers using p′ and q′ fuel strands, state II, curve (ii), and (c) upon treatment of the separated capsules, state II with the counter fuel strands p′′ and q′′ to regenerate state I, curve (iii). Error bars derived from *N* = 3 experiments.

The confocal microscopy fluorescence images of the microcapsules demonstrated the feasibility of the nucleic acid-functionalized microcapsules to form dimer structures enabling the spatial intimate contacting of the microcapsules for intercommunication of the microcapsules and operation of the biocatalytic cascade, Fig. 6(A), state I. The duplex nucleic acid bridging units generating the functional dimer microcapsule structure include, however, encoded information that allows the guided inhibition of the intercommunicated microcapsules biocatalytic cascade, Fig. 6(A). The p/q duplex generating the intercommunicated dimer dictating the biocatalytic cascade, can be separated by the addition of two fuel strands p′ and q′, state II. Thus, in the presence of the fuel strands p′ and q′, the active microcapsule configuration activating the intercommunicated biocatalytic cascade is blocked due to the separation of dimer microcapsules. That is, the blocker-induced inhibition of the biocatalytic cascade not only introduces a means to inhibit the intercell communication, but provides a proof that the intercommunication of the microcapsule originates from the intermediary formation of the dimer microcapsules. Furthermore, by the addition of counter-fuel strands p′′ and q′′ displacing the blocker units p′ and q′, the re-assembly of the active dimer structures is stimulated, resulting in the switched “ON” GOx//hemin/G-quadruplex tetrahedra biocatalytic cascade, state I. Fig. 6(B), curve (i), shows the time-dependent fluorescence changes of Resorufin formed by the intercommunicated dimer-microcapsule biocatalytic cascade driven by GOx and the hemin/G-quadruplex bridged DNA tetrahedra in state I. Fig. 6(B), curve (ii), shows the time-dependent fluorescence changes of Resorufin by the mixture consisting of the GOx-loaded microcapsules and the hemin/G-quadruplex bridged tetrahedra dimer-loaded microcapsule, treated with the fuel strands p′ and q′ and triggered by glucose. Inefficient Resorufin formation is observed, consistent with the inhibited formation of the dimer microcapsules, where the intimate spatial structure for communicating substrate transfer is perturbed. Treatment of the inhibited microcapsule mixture with the counter-fuels p′′ and q′′ reactivates the intercommunication between the microcapsule, Fig. 6(B), curve (iii). That is, the displacement of the p′/p′′ and q′/q′′ duplexes uncages the microcapsules functionalized with the p and q tethers, allowing regeneration of the intercommunicating dimer structures of the microcapsules. It should be noted that the residual very low cascaded biocatalytic activity of the p′, q′ separated GOx-loaded microcapsules and hemin/G-quadruplex-bridged T_1_/T_2_-loaded microcapsules is attributed to incomplete separation of the dimer microcapsule structure, rather than the possible exchange of loads within the dimer microcapsule structure. This is supported by an experiment, Fig. S21,† allowing the dimer-microcapsules mixture to interact for a time-interval of ten days and subsequent treatment of the mixture after this time-interval with p′, q′ to induce separation. The resulting separated microcapsule mixture demonstrated the same residual biocatalytic performance depicted in Fig. 6(B), curve (ii), indicating that no content exchange occurred even within a time-interval of ten days.

It should be noted that the rate of the biocatalytic cascade in the mixture of M_1_/M_2_ intercommunicated microcapsules is *ca.* 5-6-fold enhanced as comparted to the mixture of the homogenous biocatalysts, even though only *ca.* 9% of the microcapsules are in the “correct” configuration to allow the efficient intercommunicated cascade. This means that the “effective” rate enhancement of cascaded catalysis demonstrated by the intercommunicated pairs of microcapsules, as compared to the homogenous biocatalysts is significantly higher. Also, note that the rate enhancement by the intercommunicated microcapsules as compared to the homogenous mixture of biocatalysts is comparable to the rate enhancement demonstrated by the GOx//hemin/G-quadruplex in a single microcapsule, despite that only 9% of the microcapsules exist in the appropriate dimer configuration. This is explained by the substantially lower loading of the GOx and hemin/G-quadruplex catalysts in single microcapsules that can be accomplished in the course of the microcapsules preparation (the number of microcapsules in the different systems is approximately similar).

**Fig. 7:**
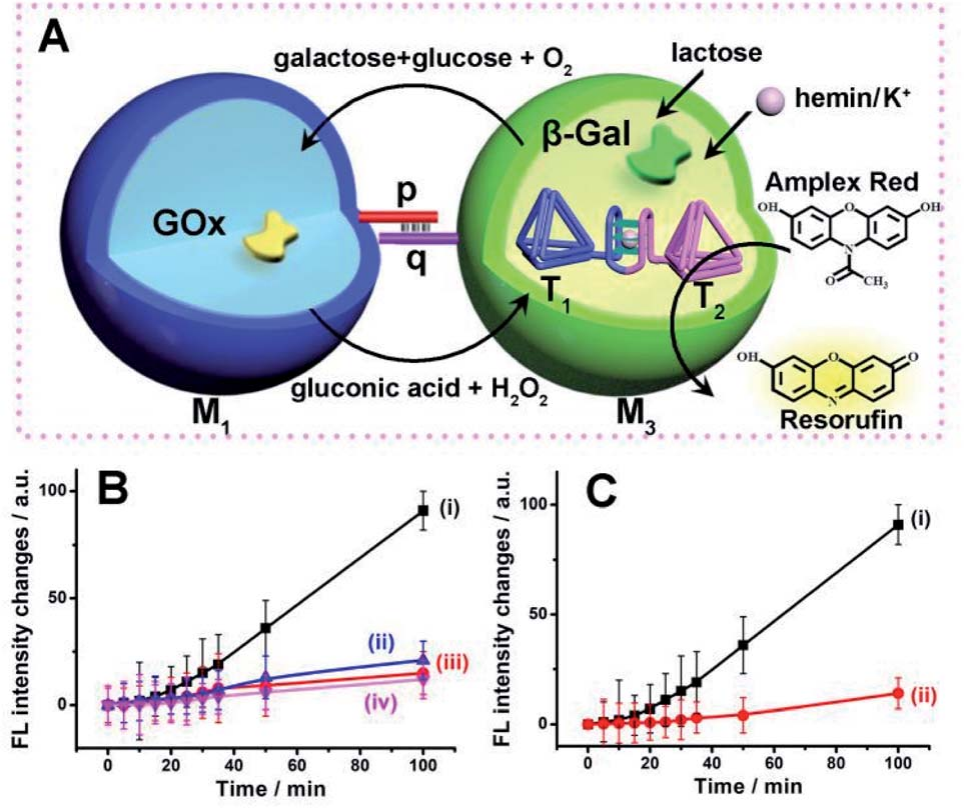
(A) Schematic intercommunication of two microcapsular microcapsules activating the three-biocatalysts cascade consisting of the lactose-driven β-gal//GOx//hemin/G-quadruplex bridged T_1_/T_2_ dimer catalytic cascade yielding the fluorescent Resorufin. (B) Time-dependent fluorescence intensities of Resorufin generated by: (i) The intercommunicated M_1_/M_3_ capsules in the presence of lactose, 40 mM, hemin, 0.167 μM, K^+^-ions, 50 mM, and Amplex Red, 0.083 mM. (ii) Lactose, 40 mM, hemin, 0.167 μM and Amplex Red, 0.083 mM, yet in the absence of K^+^-ions. (iii) As in (i), yet in the presence of CE, 200 mM. (iv) As in (i), yet in the absence of lactose. (C) Time-dependent fluorescence changes of Resorufin generated by: (i) The M_1_/M_3_ capsules, loaded with GOx, 0.12 μM, β-gal, 0.07 μM, and the hemin/G-quadruplex bridged T_1_/T_2_ dimer, 0.058 μM, in the presence of lactose, 40 mM, hemin, 0.167 μM, K^+^-ions, 50 mM, and Amplex Red, 0.083 mM. (ii) A homogeneous mixture consisting of GOx, 0.12 μM, β-gal, 0.07 μM, and the hemin/G-quadruplex bridged T_1_/T_2_ dimer, 0.058 μM, in the presence of lactose, 40 mM, hemin, 0.167 μM, K^+^-ions, 50 mM, and Amplex Red, 0.083 mM. Error bars derived from *N* = 3 experiments.

The successful operation of the biocatalyst cascade through the dynamic formation of dimer microcapsules assemblies allowed us to establish a three-catalysts cascade through the bi-directional intercommunication of the microcapsules, Fig. 7(A). The system consisted of the M_1_ microcapsules loaded with GOx, 0.12 μM, and the second type of microcapsules, M_3_-loaded with β-gal, 0.058 μM, and the hemin/G-quadruplex bridged tetrahedra T_1_/T_2_ dimer constituent, 0.07 μM. Since the microcapsular containments are functionalized with the tethers p and q, the dynamic assembly of M_1_/M_3_ dimer allows the bi-directional intercommunication of the microcapsules. In the presence of lactose, the β-gal catalyzed hydrolysis of lactose in microcapsules M_3_ yields glucose that is being transported through the dimer boundary into microcapsules M_1_. The aerobic oxidation of glucose in microcapsules M_1_ yields H_2_O_2_ that is back channeled through the dimer boundary into microcapsules M_3_, where the hemin/G-quadruplex bridged DNA tetrahedra T_1_/T_2_ catalyzes the oxidation of Amplex Red to Resorufin by H_2_O_2_. Fig. 7(B), curve (i), depicts the time-dependent fluorescence changes of Resorufin upon subjecting to two-capsule system in the presence of lactose, K^+^-ions, hemin and Amplex Red.

The three-catalysts cascade is activated through the bi-directional intercommunication of the two microcapsules. Further experiments demonstrated that exclusion of K^+^-ions from the system that resulted in the inhibition of the hemin/G-quadruplex bridged T_1_/T_2_ catalyst, prohibited the formation of Resorufin, Fig. 7(B), curve (ii). Also, addition of CE to the intercommunicating microcapsules separated the hemin/G-quadruplex bridged T_1_/T_2_ and eliminated the formation of Resorufin, Fig. 7(B), curve (iii). Finally, elimination of lactose from the system, blocked the entire intercommunicated biocatalytic cascade, Fig. 7(B), curve (iv). Fig. 7(C), compare the time-dependent fluorescence changes of Resorufin by the intercommunicated microcapsular system, curve (i), to the time-dependent fluorescence changes of Resorufin generated by the three catalysts in a homogeneous solution, where the catalysts are present at identical concentrations present in microcapsules, curve (ii). The biocatalytic cascade in the homogeneous mixture is very inefficient, and the three-catalyst cascade operating in the intercommunicating microcapsule assembly is at least 10-fold enhanced as compared to the homogeneous mixture. These results highlight the significance of bi-directional communication between the catalysts-loaded microcapsules that originates from the dynamic formation of dimer microcapsules bridged by the duplex nucleic acid p/q tethers.

## Conclusions

We introduced nucleic acid-crosslinked carboxymethyl cellulose hydrogel-stabilized microcapsules as a versatile containment for the construction of microcapsules. The microcapsule containments reveal advantages over the present art of microcapsule assemblies: (i) The hydrogel-stabilized microcapsules provide stable non-leakable containments for constituents with molecular weights, ≥5 kDa while low-molecular-weight agents freely cross the microcapsule boundaries.^65^ This allows to trigger chemical transformations in the microcapsule assemblies and to monitor the chemical transformations proceeding in the microcapsules by analyzing the chemical composition of the bulk solution. (ii) The method applied to construct the nucleic acid-modified microcapsules yields microcapsules functionalized with nucleic acid tethers revealing self-recognition properties. This unique feature provides means to yield dimer structures of two different microcapsules of programmed pre-engineered constitutional compositions. The spatial proximity of the interlinked microcapsules provide means to intercommunicate between the microcapsules and operate inter-cell catalytic cascades. (iii) The nucleic acid-guided formation of the dimer microcapsules can be switched off and re-activated by applying “fuel” and “counters-fuel” strands, thereby allowing the programmed activation and deactivation of intercommunicating microcapsule cascades. Specifically, the present study demonstrated the integration of enzymes and/or hemin/G-quadruplex-crosslinked DNA tetrahedra as biocatalysts or synthetic models for protein–protein nanostructure exhibiting catalytic (DNAzyme) activities. Besides the basic structural characterization of the catalysts-loaded microcapsules, important functionalities of the microcapsules were achieved including: (i) The integration of the hemin/G-quadruplex-crosslinked DNA tetrahedra T_1_/T_2_ dimer structure into the microcapsules and the application of the nanostructure as catalytic protein–protein model system and switchable catalytic systems. (ii) The integration of glucose oxidase and hemin/G-quadruplex crosslinked DNA tetrahedra T_1_/T_2_ DNAzyme in the microcapsules or the integration of β-galactosidase, glucose oxidase, and the hemin/G-quadruplex crosslinked DNA tetrahedra T_1_/T_2_ unit as catalytic assemblies driving bicatalyst and three-catalysts cascades. Superior catalytic cascades in the confined environments of microcapsules, as compared to the operation of the catalytic cascades in homogeneous aqueous mixtures were demonstrated. (iii) The intercommunication of microcapsules and the guided directional and bi-directional operation of bicatalytic and three-catalytic cascades were highlighted. A unique property of the nucleic acid-functionalized hydrogel microcapsules was highlighted by demonstrating the dynamic formation of dimer microcapsule assemblies that leads to spatial proximity of the microcapsules and to a mechanistic pathway to intercommunicate microcapsules.

We believe, however, that these results and the nucleic acid-based microcapsule assemblies introduce a substantially broader impact on the development of functional microcapsules; the introduction of other catalytic constituents into the microcapsules, such as DNA machineries^66^ or transcription machineries,^67^ the integration of dynamic catalytic networks^68^ into the hydrogel microcapsules, and the further functionalization of the nucleic acid-modified microcapsular hydrogel boundaries with stimuli-responsive nucleic acids that switch the chemical transformations in the microcapsules are interesting paths to follow. Furthermore, the study has emphasized the superior functions of enzyme or multi-enzyme assemblies loaded in microcapsules to drive biocatalytic transformations. Other applications of such enzyme-loaded microcapsules for therapeutic or sensing applications may be envisaged. For example, the intracapsule integration of a biocatalytic agent^69^ might yield effective carriers for drug release. Also, the amplification phenomenon of biocatalytic cascades, in the confined microcapsular environment, and the possibility to engineer nucleic acid tethers on the boundaries of the microcapsules provide means to anchor the microcapsules onto sensing recognition events as amplifying transducers. For example, by anchoring microcapsules loaded with biocatalytic cascades generating redox active compounds, to recognition events occurring on electrode surfaces, amplified electrochemical sensors can be realized.^70^

## Author contributions

P. Z., A. F. and I. W. formulated the concepts and methodology of the study. The manuscript was written through the contributions of all authors. All authors participated in the experimental work and have given approval regarding the final version of the manuscript.

## Conflicts of interest

There are no conflicts to declare.

## Supplementary Material

SC-013-D2SC01542K-s001
