# Supplementary material for: Biocatalytic cascades and intercommunicated biocatalytic cascades in microcapsule systems
Source: Chem Sci. 2022 Apr 29;13(25):7437–48. doi: 10.1039/d2sc01542k (PMC9241983; doi:10.1039/d2sc01542k)
Supplement: SC-013-D2SC01542K-s001 [file SC-013-D2SC01542K-s001.pdf]

Electronic Supplementary Material (ESI) for Chemical Science.
This journal is © The Royal Society of Chemistry 2022

Supporting Information for

Biocatalytic Cascades and Intercommunicated Biocatalytic Cascades in Microcapsule Systems

Pu Zhang,[a, c†] *Amit Fischer,*[a†] *Yu Ouyang,*[a] *Jianbang Wang,*[a] *Yang Sung Sohn,*[b] *Ola Karmi,*[b] *Rachel Nechushtai,*[b] *and Itamar Willner*[a]*

† These authors contributed equally to this work.

[a] Institute of Chemistry, Center for Nanoscience and Nanotechnology, The Hebrew University of Jerusalem, Jerusalem 91904, Israel.

[b] Institute of Life Science, The Hebrew University of Jerusalem, Jerusalem 91904, Israel.

[c] Key Laboratory of Luminescence and Real-Time Analytical Chemistry (Southwest University), Ministry of Education, College of Chemistry and Chemical Engineering, Southwest University, Chongqing 400715, People's Republic of China

E-mail: itamar.willner@mail.huji.ac.il

Materials and Methods

Materials and Instruments

Magnesium chloride, potassium chloride, calcium chloride, sodium carbonate, 2-[4-(2-Hydroxyethyl)piperazin-1-yl]ethanesulfonic acid sodium salt (HEPES), carboxymethyl cellulose (CMC, medium viscosity, D.S. 0.9), polyallylamine hydrochloride (PAH, 58 kDa), ethylenediaminetetraacetic acid disodium salt dihydrate (EDTA), Zn(II)-PPIX, β-galactosidase, β-gal, glucose oxidase, GOx, glucose, lactose, H_2O_2, Amlex Red, hemin, 18-crown-6-ether (CE), Fluorescein isothiocyanate (FITC), coumarin, N-(3-dimethylaminopropyl)-N'-ethylcarbodiimide hydrochloride (EDC), sulfo-N-hydroxysuccinimide (NHS), N, N, N', N'-tetramethy-lethylenediamine (TEMED), ammonium persulfate (APS) and acrylamide/bis-acrylamide 40% solution (suitable for electrophoresis, 19:1) were bought from Sigma-Aldrich. DNA oligonucleotides were synthesized and purified by Integrated DNA Technologies Inc. (Coralville, IA). GelRed nucleic acid gel stain was purchased from Invitrogen. Ultrapure water from NANOpure Diamond (Barnstead) source was used throughout the experiments.

A Magellan XHR 400L scanning electron microscope (SEM) and an FV-1000 confocal microscope (Olympus, Japan) and flow cytometry (CellStream Analyzer, Merck) were employed to characterize the microcapsules. Fluorescence spectra was measured with a Cary Eclipse Fluorometer (Varian Inc.). Resorufin (λ_{ex} = 560, λ_{em} = 570-700nm), Cy5 (λ_{ex} = 648 nm, λ_{em} = 668 nm), Zn(II)-PPIX (λ_{ex} = 420 nm, λ_{em} = 550-750 nm), Coumarin (λ_{ex} = 360 nm, λ_{em} = 420nm-550 nm) FITC (λ_{ex} = 490 nm, λ_{em} = 500-650 nm). The concentration of DNA oligonucleotides was standardized by UV-2401PC (SHIMADAZU) according to Beer-Lambert's Law. The gel experiment was run on a Hoefer SE 600 electrophoresis unit.

The sequences of all nucleic acid used in this study is listed as follows: (from 5' to 3'):

Promoter (1): GTAGAAGAAGGTGTCACAGTT

H_1: NH_2-$(CH_2)_6$-TTTTTTTTTTGGTGTTTAAGTTGGAGAATTGTACTTAAACACCT TCTTCT

H_2: CAATTCTCCAACTTAAACTAGAAGAAGGTGTTTAAGTTGGGCTCTAACATC GGTCCAA

(2): NH_2-$(CH_2)_6$-TTTTCTTCATTGTTT

p' (corresponding to H_1): CAA TTC TCC AAC TTA AAC GGC CGT

p'': ACG GCC-GTT TAA GTT GGA GAA TTG

q' (corresponding to H_2): CCT GGC -CTT AAA CAC CTT CTT CT

q'' (corresponding to H_2): AG AAG AAG GTG TTT AAG-GCC AGG

S_1: ACACTACGTCAGAACAGCTTGCATCACTGGTCACCAGAGTA

S_1-Cy5: Cy5-ACACTACGTCAGAACAGCTTGCATCACTGGTCACCAGAGTA

S_1-Cy3: Cy3-ACACTACGTCAGAACAGCTTGCATCACTGGTCACCAGAGTA

S_2: TCAACTCGCTCGTAACTACACTGTGCAATACTCTGGTGACC

S_3: TCTGACGTAGTGT ATGCACAGTGTAGTAAGGACCCTCGCAT

S_4:

CTCGTGTTTTACACTACGTCAGAACAGCTTGCATCACTGGTCACCAGAGTATTTTT GGGGAATGGGGTTTGAGTGCC

S_5:

GGCACTCTTTGGGGAATGGGGTTTTTACACTACGTCAGAACAGCTTGCATCACTG GTCACCAGAGTATTTTCACGAG

Tetrahedra T_1 is consisted of (S_1) (S_2) (S_3) and (S_4), Cy5-labeled tetrahedra T_1 is consisted of (S_1-Cy5) (S_2) (S_3) and (S_4). Tetrahedra T_2 is made of (S_1) (S_2) (S_4) and (S_5), Cy3-labeled tetrahedra T_2 is consisted of (S_1-Cy3) (S_2) (S_4) and (S_5). The sequence of the G-quadruplex is underlined.

Methods

Construction of DNA tetrahedra nanostructure.

The DNA tetrahedra, used in this study, consisted of four sequences, were prepared as follows. A mixture of (S_1), (S_2), (S_3) and (S_4) (or a mixture of (S1) (S2) (S3) and (S5)) (2 µM each) in 10 mM HEPES buffer (containing 20 mM $MgCl_2$, pH = 7.2) was annealed at 95 °C for 5 min, subsequently, cooled down to 4 °C, and allowed to equilibrate at 25 °C for 2 hours, yielding DNA tetrahedra T_1 or T_2. The tetrahedra DNA nanostructures were characterized by native PAGE and AFM.

Synthesis of 5'-Amino Modifier C_6-modified oligo/Carboxymethyl cellulose (CMC) copolymers

2 mL of a MES buffer solution (10 mM, pH 5.5), containing CMC, 20 mg, N-(3-dimethylaminopropyl)-N'-ethylcarbodiimide hydrochloride (EDC), 20 mg, were incubated for 5 minutes and then sulfo-N-hydroxysuccinimide (NHS), 26 mg, was added, and the solution was incubated for additional 10 minutes. To the resulting solution, 2 mL of HEPES buffer (50 mM, pH 7.2) containing the amine-functionalized nucleic acids (900 µM of H_1 for P_1 or 900 µM of (**2**) for P_2), were added. The mixture was gently shaked for 2 h at room temperature.

The modified polymers, P_1 (modified with H_1) and P_2 (modified with (**2**)), were purified and separated from the unreacted compounds using MWCO 10K Amicon spin filters. After being washed with water for three times, the copolymer solutions were dried and re-dispersed in buffer (10 mM HEPES, pH 7.0, containing 25 mM $MgCl_2$). To polymer P_2, after the determination of the concentration of (**2**), hairpin H_2 was added in a molar ratio of 1:1. The polymer solutions were incubated at 95 °C for 5 min, followed immediately by incubation on ice for 30 min to ensure the efficient closing of the hairpins. All the prepared samples were keep in 4 °C for further use.

Preparation of $CaCO_3$ microparticles with different loads

$CaCO_3$ particles were prepared by a precipitation reaction between equal amounts of $CaCl_2$ and Na_2CO_3 under magnetic stirring at room temperature. $CaCO_3$ particles loaded with tetrahedra T_1/T_2 were obtained through co-precipitation by mixing $CaCl_2$ (307 μL, 0.33 M) and Na_2CO_3 (307 μL, 0.33 M) solutions, in the presence of tetrahedra T_1/T_2 (30 μL, 4 μM of each). (For quantification, the $CaCO_3$ particles were loaded with Cy5-labeled T_1, 4 μM, 30 μL, and unlabeled T_2). The final volume was adjusted to 1020 μL by addition of deionized water. After magnetic stirring for 110 s, 700 rpm, the suspension was left for 70s at room temperature to settle down. The particles were centrifuged at 100 rcf for 30s, followed by the removal of the supernatant solution, and the subsequent resuspension of the particles in water. This washing procedure was repeated twice in order to remove the byproducts resulting from the precipitation reaction.

For 2-enzyme cascade: GOx or coumarin-labeled GOx: 30 μL, 10 mg mL^{-1}, T_1 and T_2 or Cy5-labeled T_1, 4 μM, 30 μL. For 3-enzyme cascade: β-gal or FITC-labeled β-gal 15 μL, 10 mg mL^{-1}; GOx or coumarin-labeled GOx: 15 μL, 10 mg mL^{-1}, T_1 and T_2 or Cy5-labeled T_1, 4

μM, 30 μL. For intercommunication: M_1 was loaded with GOx or coumarin-labeled GOx: 30 μL, 10 mg mL^{-1}, M_2 was loaded with T_1 and T_2 or Cy5-labeled T_1, 4 μM, 30 μL, M_3 was loaded with β-gal, 15 μL, 10 mg mL^{-1}, T1 and T_2 or Cy5-labeled T_1, 4 μM, 30 μL).

Synthesis of DNA-based CMC hydrogel microcapsular microcapsules

The $CaCO_3$ microparticles were suspended in 600 μL of 1 mg mL^{-1} PAH solution (10 mM HEPES, pH 7.2, containing 25 mM $MgCl_2$) and kept under continuous shaking for 30 min. The PAH-coated particles were washed twice with buffer (10 mM HEPES, pH 7.2, containing 25 mM $MgCl_2$), followed by centrifugation at 100 rcf for 30 s. Subsequently, the PAH-coated microparticles were incubated with 600 μL of the promoter nucleic acid (**1**) (final concentration 10 μM) and kept under continuous shaking at room temperature for 30 min. After being washed twice with buffer (10 mM HEPES, pH 7.2, containing 25 mM $MgCl_2$), followed by centrifugation at 100 rcf for 30 s, the DNA hydrogel particles were prepared by mixing the polymer sets (P_1/P_2) with the promoter-coated $CaCO_3$ microparticles. The final concentration of each hairpin was 10 μM. The particles were incubated overnight (approximately 12h) at room temperature under continuous shaking, followed by centrifugation at 100 rcf for 30 s to remove non-adsorbed polymers and the subsequent resuspended in buffer (10 mM HEPES, pH 7.0, containing 25 mM $MgCl_2$). This washing procedure was repeated twice.

120 μL of a 0.5 M EDTA solution (pH 7.5) were added into 60 μL of microparticle solution containing 4500 microcapsules/μL and 60 μL of buffer solution (10 mM HEPES, pH 7.2, containing 25 mM $MgCl_2$). The resulting solution was incubated for 0.5 h to dissolve the $CaCO_3$ cores. When the suspension became clear, the capsules were washed with buffer (10 mM HEPES, pH 7.2, containing 25 mM $MgCl_2$) for three times (50 rcf, 20 min).

The preparation of the different cargo-loaded microcapsules is fully reproducible with no effect of the cargo integrated in the microcapsules. Within a set of five repeated experiments of different microcapsules described in the study, the results presented in the paper were reproduced with an accuracy corresponding to ± 6%.

Catalysis and cascaded catalysis in microcapsules

To explore catalytic properties of microcapsules that loaded with tetrahedra T_1 and T_2 (depicted in Figure 2 (A)), hemin, 0.167 μM, and K^+ (50 mM) were added to the microcapsule solution, followed by incubation at room temperature for 0.5 hour. H_2O_2, 4.16 mM and Amplex Red, 0.083 mM, were added to the above mixture. The fluorescence emission spectra of Resorufin was collected from 570 to 700 under excitation of 560 nm. After a fixed time-interval, 200 mM of CE was introduced to the system to separate the G-quadruplex-bridged tetrahedra dimer T_1/T_2. Finally, the microcapsules were washed by Tris buffer containing 50 mM K^+-ions and resuspended in Tris buffer containing 50 mM K^+-ions.

To demonstrate the formation of G-quadruplex-bridged tetrahedra dimer T_1/T_2 by Zn(II)-PPIX, the microcapsules loaded with tetrahedra T_1 and T_2 (depicted in Figure 2 (B)), was subjected to and K^+ (50 mM) and Zn(II)-PPIX (2 μM), followed by incubation at room temperature for 0.5 hour. The fluorescence emission spectra of Zn(II)-PPIX was collected from 550 to 750 under excitation of 420 nm. After a fixed time-interval, 200 mM of CE was introduced to the system to separate the G-quadruplex-bridged tetrahedra dimer T_1/T_2. Finally, the microcapsules were washed by Tris buffer containing 50 mM K^+-ions and resuspended in Tris buffer containing 50 mM K^+-ions.

To explore catalytic properties of microcapsules that loaded with tetrahedra T_1 and T_2 and GOx (depicted in Figure 3 (A)), glucose, 10 mM, hemin, 0.167 μM, and K^+ (50 mM) were added to the microcapsule solution, followed by incubation at room temperature for 0.5 hour.

Amplex Red, 0.083 mM, were added to the above mixture. The fluorescence emission spectra of Resorufin was collected from 570 to 700 under excitation of 560 nm. After a fixed time-interval, 200 mM of CE was introduced to the system to separate the G-quadruplex-bridged tetrahedra dimer T_1/T_2.

To study catalytic properties of microcapsules that loaded with tetrahedra T_1 and T_2, GOx and β-gal, (depicted in Figure 4 (A)), lactose, 40 mM, hemin, 0.167 μM, and K^+ (50 mM) were added to the microcapsule solution, followed by incubation at room temperature for 0.5 hour. Amplex Red, 0.083 mM, were added to the above mixture. The fluorescence emission spectra of Resorufin was collected from 570 to 700 under excitation of 560 nm. After a fixed time-interval, 200 mM of CE was introduced to the system to separate the G-quadruplex-bridged tetrahedra dimer T_1/T_2.

Intercommunication between microcapsules

To study the intercommunication between microcapsules M_1 and M_2 (depicted in Figure 6 (A)), glucose, 30 mM, hemin, 0.167 μM, and K^+ (50 mM) were added to the mixture of M_1/M_2 solution, followed by incubation at room temperature for 0.5 hour. Amplex Red, 0.083 mM, were added to the above mixture. The fluorescence emission spectra of Resorufin was collected from 570 to 700 under excitation of 560 nm. After a fixed time-interval, 200 mM of CE was introduced to the system to separate the G-quadruplex-bridged tetrahedra dimer T_1/T_2.

To study the intercommunication between microcapsules M_1 and M_3 (depicted in Figure 9 (A)), lactose, 40 mM, hemin, 0.167 μM, and K^+ (50 mM) were added to the mixture of M_1/M_3 solution, followed by incubation at room temperature for 0.5 hour. Amplex Red, 0.083 mM, were added to the above mixture. The fluorescence emission spectra of Resorufin was collected from 570 to 700 under excitation of 560 nm. After a fixed time-interval, 200 mM of CE was introduced to the system to separate the G- quadruplex-bridged tetrahedra dimer T_1/T_2.

For the switchable intercommunication between microcapsules M_1 and M_2 (depicted in Figure 8), 0.5 mM of p' and q' was subjected to the mixture of M_1/M_2 protcells, followed by incubated for 6h before measure the cascaded catalysis. Furthermore, 1 mM of p'' and q'' was subjected to the above mixture of M_1/M_2 protcells containing p' and q', followed by incubated for 6h before measure the cascaded catalysis. glucose, 30 mM, hemin, 0.167 μM, and K^+ (50 mM) were added to the mixture of M_1/M_2 solution, followed by incubation at room temperature for 0.5 hour. Amplex Red, 0.083 mM, were added to the above mixture. The fluorescence emission spectra of Resorufin was collected from 570 to 700 under excitation of 560 nm.

Confocal microscopy measurements

10 μL of each sample (4500 microcapsules/μL) was loaded on a microscopic glass slide with coverslips. Fluorescent images of microcapsules with filter set were recorded using confocal microscopy (the Olympus FV3000 confocal laser-scanning microscope) and all images were analyzed with image J for analyzing microcapsules as follows. Microcapsules, from ten different fluorescence domains of three different mixture samples (total 30 field frames), were counted as monomer, dimer and undefined objects by size and color using analyzing particles by Image J program. Results were presented as percentage of defined or undefined capsules in total number of capsules.

Coumarin channel: E_x=405 nm, E_m=430-470 nm

Cy5 channel: E_x=640 nm, E_m=650-700 nm

FACS analysis

Microcapsules were quantitatively investigated with the flow cytometry (CellStream Analyzer, Merck). The mixture of coumarin-labeled GOx-loaded capsules and Cy5-labeled T_1/T_2-loaded microcapsules were introduced

into FACS and 100k microcapsule events were recorded of each measurement. Percentage of different microcapsule population and quantitative microcapsule counts were computed and analyzed with Cell Stream acquisition and analysis software. Results are presented as box and whiskers plots. All data points were measured by three different samples.

Coumarin channel: E_x=405 nm, E_m=456 nm

Cy5 channel: E_x=642 nm, E_m=702 nm

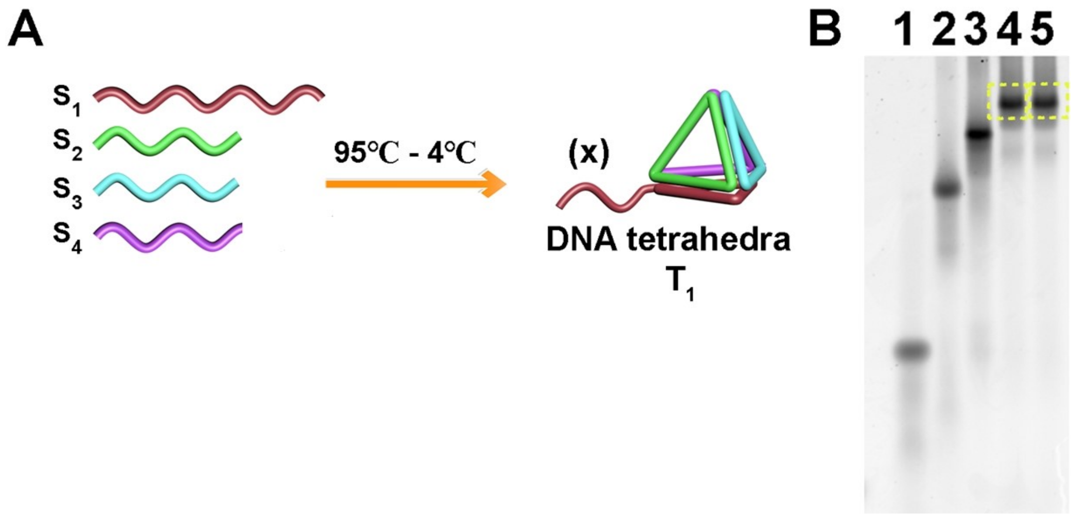

Figure S1 (A) Schematic assembly of the DNA tetrahedra T_1 using the strands S_1-S_4. The tether (x) is half G-quadruplex sequence. (B) Electrophoretic characterization of the DNA tetrahedra T_1 and effect of EDTA upon etching the $CaCO_3$ core on which the tetrahedra nanostructures are deposited. (Electrophoretic separation on 12% PAGE gel). Lane 1-Strand S_1; Lane 2-S_1+S_2; Lane 3-S_1+S_2+S_3; Lane 4-Intact tetrahedra consisting of S_1+S_2+S_3+S_4 prior to the deposition on $CaCO_3$ particles; Lane 5-Intact tetrahedra (with no fragmented strands) upon analysis of the solution obtained after EDTA etching, 0.1 M, of the $CaCO_3$ particles impregnated with the tetrahedra T_1.

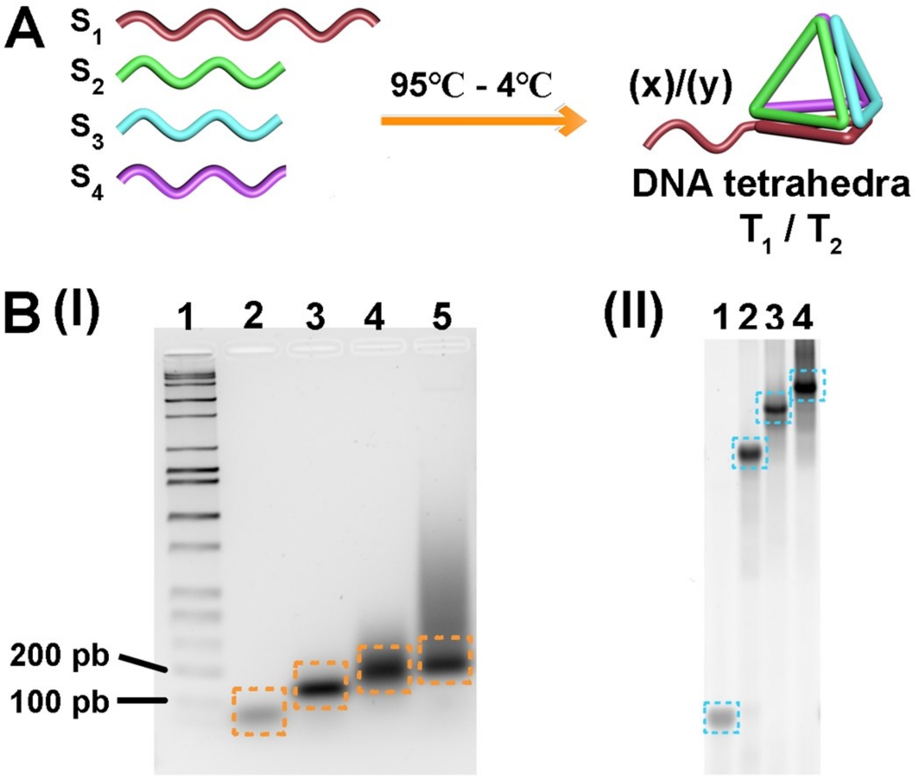

Figure S2 (A) Schematic assembly of the DNA tetrahedra T_1 or T_2 using the four strands. The tether (x)/(y) is half G-quadruplex sequence. (B) Panel (I)-Electrophoretic separation on 1% agarose gel following the stepwise assembly of tetrahedra T_1 nanostructure: Lane 1-Ladder; Lane 2-S_1; Lane 3-S_1+S_2; Lane 4-S_1+S_2+S_3; Lane 5-S_1+S_2+S_3+S_4. Panel (II)-Electrophoretic separation on 12% PAGE gel following the stepwise assembly of tetrahedra T_2 nanostructure: Lane 1-S_1; Lane 2-S_1+S_2; Lane 3-S_1+S_2+S_4; Lane 4-S_1+S_2+S_4+S_5.

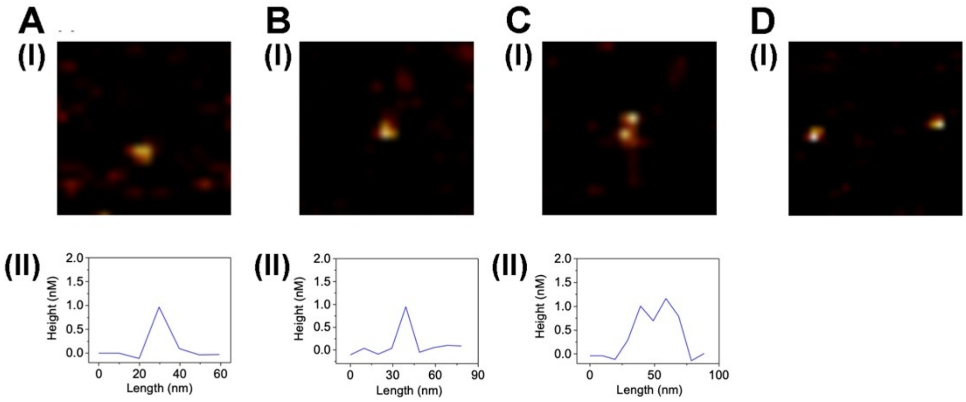

Figure S3 (A) Panel I-AFM image corresponding to T_1. Panel II-Cross-section analysis of the height corresponding to T_1 DNA tetrahedra. (B) Panel I-AFM image corresponding to T_2. Panel II-Cross-section analysis of the height corresponding to T_2 DNA tetrahedra. (C) Panel I-AFM image corresponding to K^+-ions stabilized G-quadruplex bridged tetrahedra dimers T_1/T_2. Panel II-Cross-section analysis of the height corresponding to K^+-ions stabilized G-quadruplex bridged tetrahedra dimers T_1/T_2. (D) AFM image corresponding to G-quadruplex bridged tetrahedra dimers T_1/T_2 before the treatment of K^+-ions.

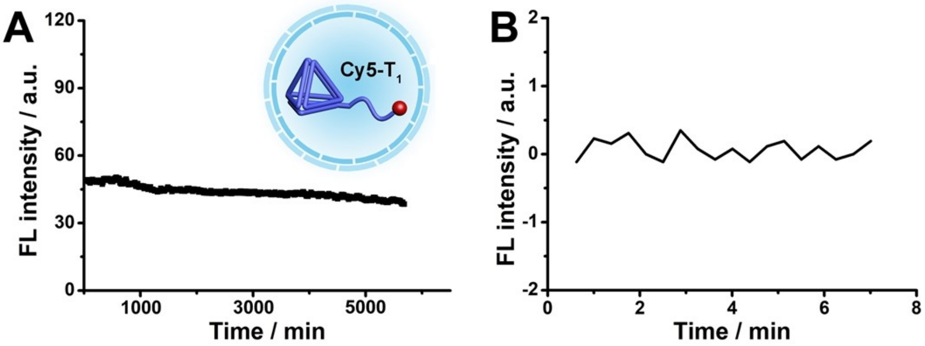

Figure S4 Time-dependent fluorescence intensity of (A) Cy5-labeled T_1 microcapsular microcapsule and (B) the supernatant of the Cy5-labeled T_1-loaded microcapsular microcapsule after washing. No leakage of the tetrahedra or their constituents from the microcapsule containments could be detected within this time-interval. It should be noted that the supernatant of the microcapsules is a homogeneous phase, the fluorescence intensity of the supernatant does not change over time.

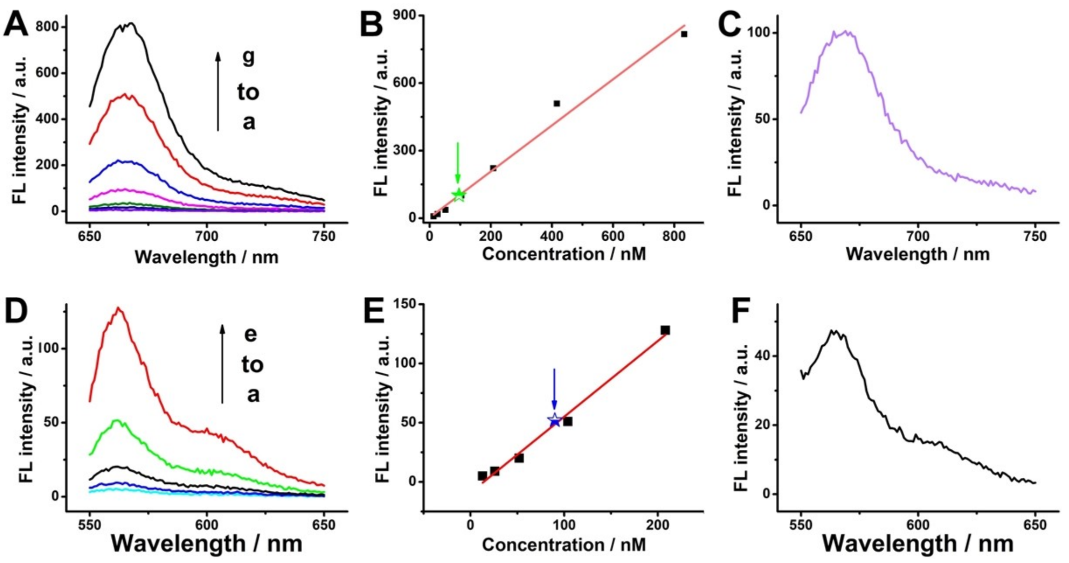

Figure S5. (A) Fluorescence spectra upon different concentrations of Cy5-labeled T_1: (a) 0.013 uM; (b) 0.026 uM; (c) 0.052 uM; (d) 0.10 uM; (e) 0.21 uM; (f) 0.42 uM; (g) 0.83 uM. (B)The calibration curve corresponding to the fluorescence intensities as a function of the concentration of Cy5-labeled T_1-loaded microcapsular microcapsule. The fluorescence intensity of Cy5-labeled T_1 was marked in arrow. (C) The fluorescence spectrum of Cy5-labeled T_1. The loading of the tetrahedra T_1 and T_2 in the microcapsules was evaluated to be 0.1 uM by using appropriate calibration curves with labeling T_1 with Cy5. (D) Fluorescence spectra upon different concentrations of Cy3-labeled T_2: (a) 0.013 uM; (b) 0.026 uM; (c) 0.052 uM; (d) 0.104 uM; (e) 0.208 uM. (E)The calibration curve corresponding to the fluorescence intensities as a function of the concentration of Cy3-labeled T_2-loaded microcapsular microcapsule. The fluorescence intensity of Cy3-labeled T_2 was marked in arrow. (F) The fluorescence spectrum of Cy3-labeled T_2. The loading of the tetrahedra T_1 and T_2 in the microcapsules was evaluated to be 0.09 uM by using appropriate calibration curves with labeling T_2 with Cy3.

For quantify the loading of T_1, the microcapsule was loaded with Cy5-labeled T_1 and unlabeled T_2. For quantify the loading of T_2, the microcapsule was loaded with unlabeled T_1 and Cy3-

labeled T_2.

Operation of Zn(II)-PPIX intercalated tetrahedra T_1/T_2 in the microcapsule system

Previous studies demonstrated that binding of Zn(II) protoporphyrin IX, Zn(II)-PPIX, to G-quadruplex structures yields highly fluorescent complexes (Zn(II)-PPIX alone exhibits low fluorescence). Accordingly, the microcapsules, loaded with the tetrahedra T_1 and T_2, functionalized with G-quadruplex subunits, were subjected to Zn(II)-PPIX and K^+-ions to yield the fluorescent Zn(II)-PPIX-loaded G-quadruplex bridged tetrahedra T_1/T_2 dimer, Figure S6. The subsequent addition of CE, separates the G-quadruplex and results in the formation of the single tetrahedra nanostructures. Figure S6, panel I shows the fluorescence features of the Zn(II)-PPIX and T_1, T_2-loaded microcapsules, curve (a). Very low fluorescence of Zn(II)-PPIX is observed. Subjecting the microcapsules to K^+-ions induces the formation of the G-quadruplex bridged tetrahedra dimer T_1/T_2, where Zn(II)-PPIX binds to the G-quadruplex, leading to a highly fluorescent tetrahedra dimer nanostructure, Figure S6, curve (b). Treatment of the system with CE, separates the G-quadruplex and the formation of the dimer system, revealing very low fluorescence, curve (c). Subjecting the system with additional K^+-ions, the G-quadruplex bridged tetrahedra dimer T_1/T_2 was regenerated and high fluorescence intensity of Zn(II)-PPIX is observed, curve (d). By the cyclic addition of K^+-ions and CE, the reversible switched "ON"/"OFF" Zn(II)-PPIX/G-quadruplex fluorescence of the bridged T_1/T_2 supramolecular structure is observed, Figure S6, panel II. From the fluorescence spectra of the Zn(II)-PPIX/G-quadruplex bridged T_1/T_2, and using an appropriate calibration curve, Figure S7, the loading of the T_1/T_2 dimers in the microcapsules was evaluated to be 0.86 μM. (For confocal microscopy images of the microcapsules loaded with Zn(II)-PPIX/G-quadruplex bridged T_1/T_2 tetrahedra before the addition of K^+-ions and after the addition of K^+-ions, see Figure S8).

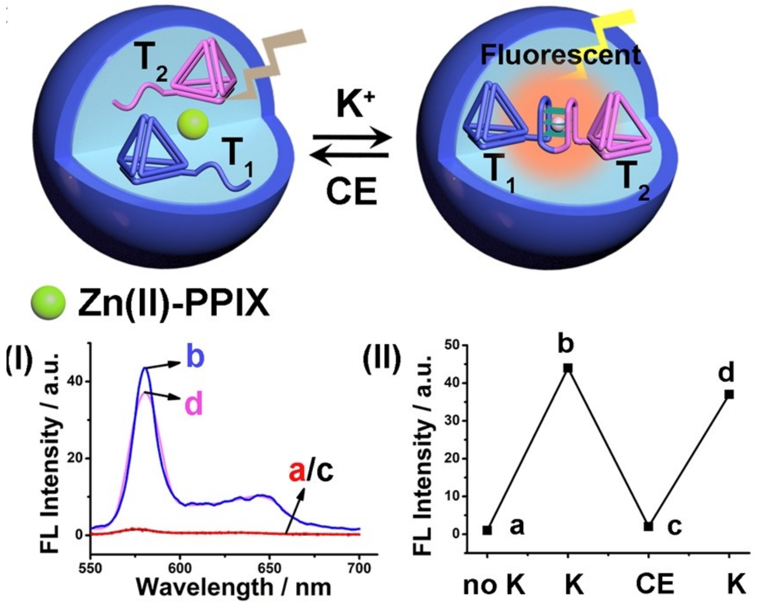

Figure S6. Cyclic K^+-ions stimulated dimerization of the DNA tetrahedra T_1 and T_2 by G-quadruplex and their separation by means of 18-crown-6-ether (CE). Formation of the K^+-stabilized G-quadruplex is probed by the fluorescence of Zn(II)-PPIX. Fluorescence spectra of the microcapsules that includes Zn(II)-PPIX, 2 µM: Panel I-In the presence of the T_1/T_2-loaded microcapsules. (b) After the addition of K^+-ions, 50 mM, allowing the formation of the G-quadruplex-bridged T_1/T_2 dimer and the generation of fluorescent Zn(II)-PPIX/G-quadruplex complex. (c) In the presence of K^+-ions, 50 mM, and treatment of CE, 200 mM. (d) After the addition of K^+-ions, 50 mM, and treatment of CE, 200 mM, 300 mM of K^+-ions was re-added to the bulk solution, and allowing Zn(II)-PPIX to bind to G-quadruplex bridged T_1/T_2 tetrahedra dimer for a time-interval of 40 minutes. Panel II-Switchable fluorescence intensities of Zn(II)-PPIX: (a) and (c) In the presence of the microcapsules loaded with the separated tetrahedra T_1 and T_2. (b) and (d) In the presence of the K^+-ions stabilized G-quadruplex bridged T_1/T_2 tetrahedra dimer.

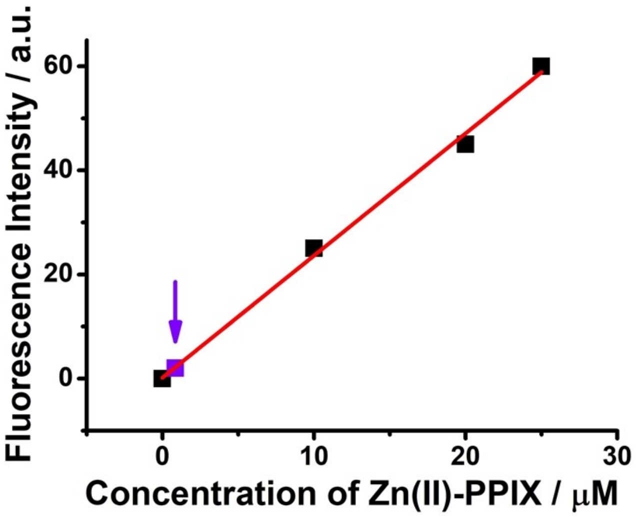

Figure S7 The calibration curve corresponding to the fluorescence intensities as a function of the concentration of Zn(II)-PPIX. The microcapsular microcapsules loaded with Zn(II)-PPIX that bind to K^+-ions stabilized G-quadruplex bridged T_1/T_2 was marked in arrow.

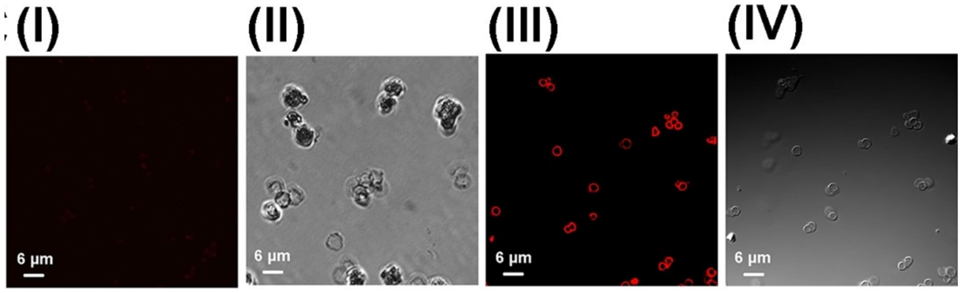

Figure S8 Confocal microscopy images of T_1/T_2-loaded microcapsular microcapsules treated with Zn(II)-PPIX in the absence of K^+-ions: (I) dark field and (II) bright field. Confocal microscopy images of the fluorescent Zn(II)-PPIX/G-quadruplex bridged T_1/T_2 tetrahedra in the presence of K^+-ions, 50 mM: (III) dark field and (IV) bright field.

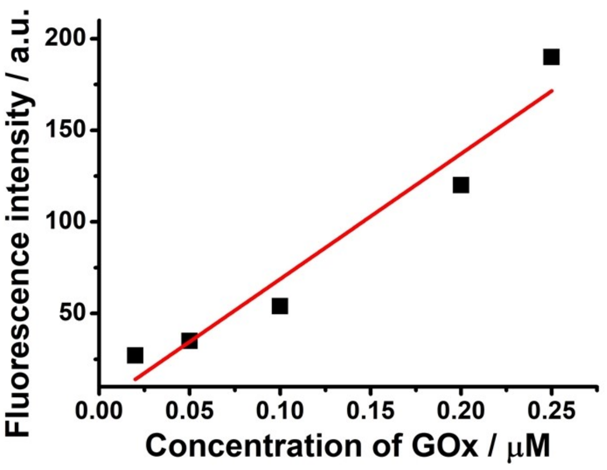

Figure S9 The calibration curve corresponding to the fluorescence intensities as a function of the concentration of coumarin-labeled GOx.

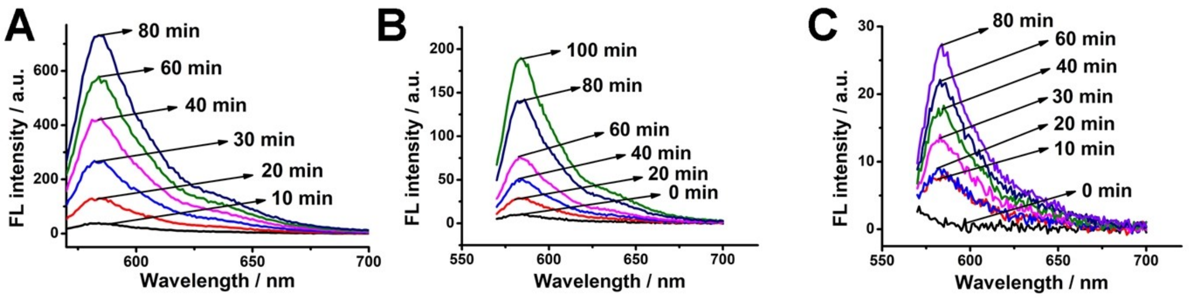

Figure S10 The fluorescence spectra of Resorufin at different time-intervals upon treatment the microcapsules with different concentrations of glucose: (A) 10 mM of glucose. (B) 2 mM of glucose. (C) In the absence of glucose.

Operation of a three biocatalyst cascade in the microcapsule system

The successful activation of the biocatalyst cascade in the microcapsular microcapsule was followed by the operation of a three-biocatalyst cascade in the microcapsular microcapsules. The three biocatalysts encapsulated in the microcapsule included β-galactosidase, β-gal, GOx and the hemin/G-quadruplex DNAzyme-bridged tetrahedra T_1/T_2 nanostructure, Figure S11 (A). By labeling the β-gal with fluorescein isothiocyanate (FITC), GOx with Coumarin, and T_1/T_2 with Cy5, the concentrations of β-gal, GOx and hemin/G-quadruplex dimer tetrahedra T_1/T_2 in the microcapsules were evaluated to be 0.037 μM, 0.035 μM and 0.004 μM, respectively. (The calibration curve of FITC-labeled β-gal is shown in Figure S12) It should be noted that the three-biocatalysts loaded microcapsule represent stable microstructure. No leakage of the loaded constituents from the microcapsules are observed for a time-interval of ten-days (Figure S13). In the presence of lactose and Amplex Red, the three-biocatalyst cascade shown in Figure S11 (A) proceeds in the microcapsule, confined containment. The β-gal catalyzed hydrolysis of lactose yields glucose that is channeled to GOx catalyzing the aerobic oxidation of glucose to form gluconic acid and H_2O_2, where the resulting H_2O_2 acts as substrate for the hemin/G-quadruplex DNAzyme that catalyzes the oxidation of Amplex Red to Resorufin. The fluorescence of the resulting Resorufin provides the readout signal for the three biocatalysts cascade. It should be noted that related three biocatalytic cascades were previously reported in other confined microenvironments.[113] The novelty of the present study rests, however, on the performance of the biocatalytic cascade in the DNA-based hydrogel microcapsular containments that allows us not only to probe by the three-biocatalysts as a model system for biocatalytic cascades in microcapsular microcapsule environments, but also to apply this biocatalytic constituents for modelling inter-capsule interactions emulating cell-cell interactions, *vide infra*. Figure S11 (B), curve (i), depicts the time-dependent fluorescence changes of Resorufin resulting in upon operation of the three-biocatalyst cascade in the

microcapsular microcapsules in the presence of K^+-ions. Control experiments revealed that in the absence of K^+-ions, no oxidation of Amplex Red proceeds, curve (ii). Addition of CE to the system treated with K^+-ions, prohibits the formation of Amplex Red, consistent with the CE-induced separation of the K^+-ions stabilized hemin/G-quadruplex DNAzymes, a process that switches off the biocatalytic cascade. Also, all biocatalytic components are essential to induce the catalytic cascade, and exclusion of any of the catalytic constituents prohibits the formation of Amplex Red. Figure S14 shows the fluorescence spectra of Resorufin at different time-intervals upon treatment the microcapsules in the presence of lactose or in the absence of lactose. Figure S15 shows the time-dependent fluorescence changes of Resorufin upon different concentrations of lactose. The three-biocatalysts assembly reveal, as expected, enhanced catalytic performance as compared to a homogeneous mixture of the catalysts that includes the three-biocatalysts at similar concentrations as present in the microcapsules, Figure S11 (C). The three-biocatalyst cascaded in the confined microcapsular microcapsule environment (curve (i)) reveal a *ca.* 5-fold enhanced activity as compared to the biocatalytic cascade driven by the three-biocatalysts in the homogeneous mixture, curve (ii). Thus, the efficient products/substrates channeling in the confined microcapsule environment leads to the effective biocatalytic cascade.

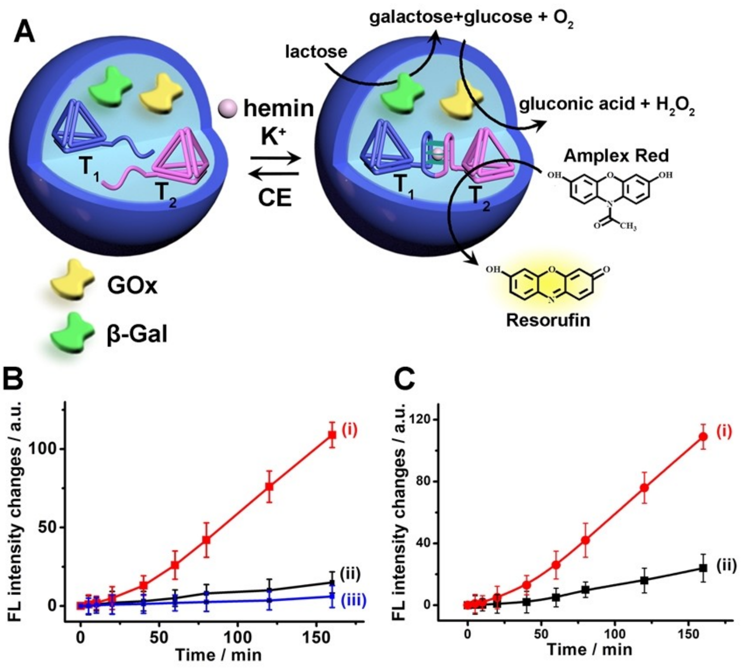

Figure S11 (A) Schematic operation of a switchable catalytic cascade consisting β-galactosidase, β-gal, glucose oxidase, GOx, and hemin/G-quadruplex bridged tetrahedra dimer T_1/T_2 loaded in microcapsular microcapsule and b-gal catalyzed hydrolysis of lactose yields glucose that is channeled to GOx catalyzing the aerobic oxidation of glucose to form gluconic acid and H_2O_2, where the resulting H_2O_2 acts as substrate for the hemin/G-quadruplex DNAzyme that catalyzes the oxidation of Amplex Red to Resorufin. (B) Time-dependent fluorescence changes of Resorufin generated by: (i) The microcapsules loaded with β-gal, GOx and the separated tetrahedra, T_1 and T_2 in the presence of lactose, 40 mM, K^+-ions, 50 mM, Amplex Red, 0.083 mM, hemin, 0.167 µM. (ii) The microcapsules loaded with β-gal, GOx and the separated tetrahedra, T_1 and T_2 in the presence of lactose, 40 mM, Amplex Red, 0.083 mM, hemin, 0.167 µM, in the absence of K^+-ions. (iii) After the addition of K^+-ions, 50 mM, the system was treated with CE, 200 mM, and allowing the separation of hemin/G-quadruplex bridged T_1/T_2 tetrahedra dimer. (C) Time-dependent fluorescence of Resorufin generated by: (i) The β-gal//GOx//hemin/G-quadruplex bridged tetrahedra T_1/T_2 confined to the microcapsular microcapsules. (ii) The β-gal//GOx//hemin/G-quadruplex constituents in a

homogeneous phase at the same concentrations of the catalysts present in the microcapsules. β-gal, 0.037 μM, GOx, 0.035 μM, hemin/G-quadruplex bridged tetrahedra T_1/T_2 dimer 0.004 μM, lactose, 40 mM, Amplex Red, 0.083 mM, hemin, 0.167 μM and K^+-ions 50 mM. Error bars derived from $N = 3$ experiments.

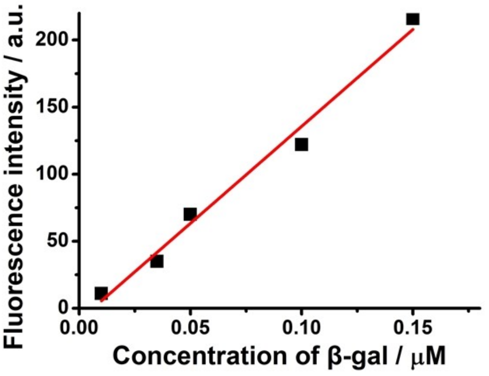

Figure S12 The calibration curve corresponding to the fluorescence intensities as a function of the concentration of FITC-labeled β-gal.

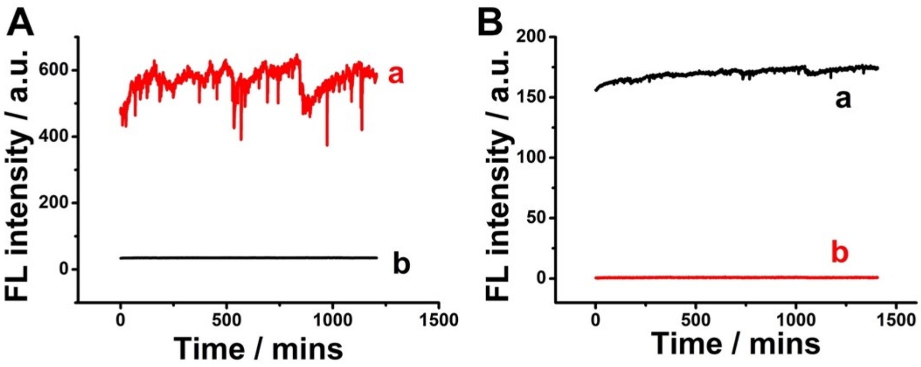

Figure S13 (A) Time-dependent fluorescence intensity of FITC-labeled β-gal-loaded microcapsular microcapsule, curve (a) and (b) the supernatant of the FITC-labeled β-gal-loaded microcapsular microcapsule after washing. No leakage of the β-gal from the microcapsule containments could be detected within this time-interval. (B) Time-dependent fluorescence intensity of coumarin-labeled GOx-loaded microcapsular microcapsule, curve (a) and (b) the supernatant of the coumarin-labeled GOx-loaded microcapsular microcapsule after washing.

No leakage of the GOx from the microcapsule containments could be detected within this time-interval.

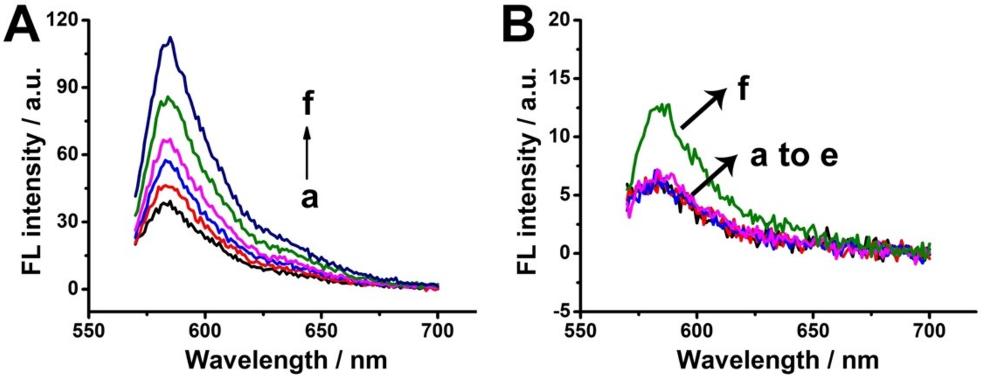

Figure S14 Fluorescence spectra of Resorufin at different time-intervals upon treatment the β-gal, GOx and the hemin/G-quadruplex DNAzyme-bridged tetrahedra loaded microcapsules in the presence of (A) 40 mM lactose; (B) In the absence of lactose at different time-intervals: (a) 20 mins; (b) 45 mins; (c) 60 mins; (d) 75 mins; (e) 125 mins; (f) 150 mins.

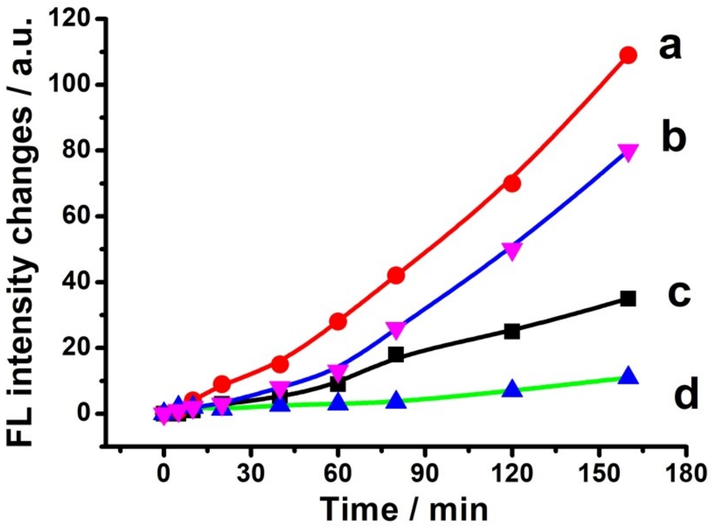

Figure S15 Time-dependent fluorescence intensity of Resorufin upon treatment the β-gal, GOx and the hemin/G-quadruplex DNAzyme-bridged tetrahedra loaded microcapsules with different concentrations of lactose: (a) 40 mM; (b) 30 mM; (b) 20 mM; (d) 0 mM.

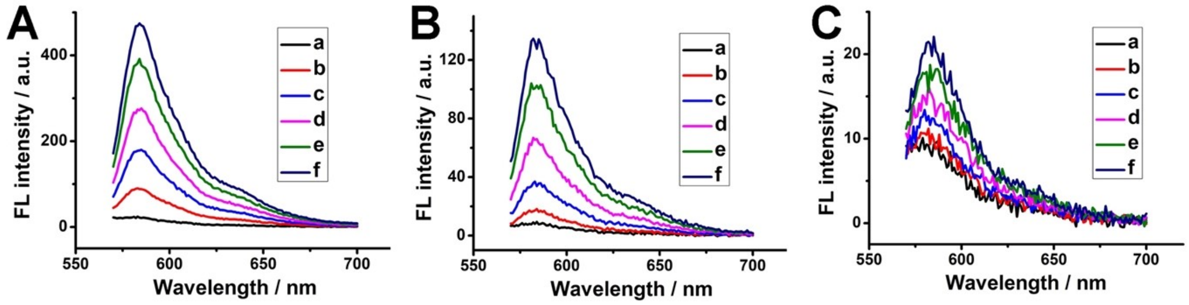

Figure S16 Fluorescence spectra of Resorufin at different time-intervals upon treatment the M_1/M_2 microcapsule mixture, β-gal-loaded microcapsules, and GOx//hemin/G-quadruplex DNAzyme-bridged tetrahedra T_1/T_2-loaded microcapsules at different time-intervals in the presence of (A) 30 mM glucose; (B) 10 mM glucose and (C) in the absence of glucose upon different time-intervals: (a) 0 mins; (b) 10 mins; (c) 15 mins; (d) 30 mins; (e) 40 mins; (f) 45 mins.

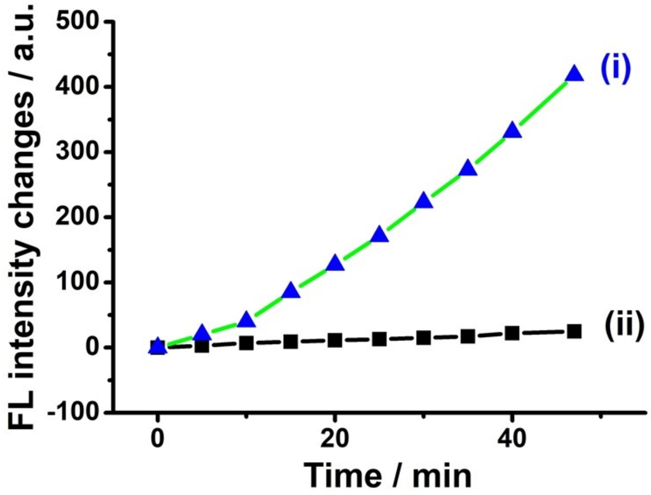

Figure S17 Time-dependent fluorescence intensity of Resorufin upon treatment the M_1/M_2 microcapsule mixture, GOx-loaded microcapsules, and hemin/G-quadruplex DNAzyme-bridged tetrahedra T_1/T_2-loaded microcapsules after three days. (i) The microcapsules resuspended in the buffer solution containing K^+-ions, 50 mM, glucose 30 mM and Amplex Red 0.083 mM. (ii) The supernatant of the M_1/M_2 microcapsule mixture with the addition of K^+-ions, 50 mM, glucose 30 mM and Amplex Red 0.083 mM.

Control experiments revealed that no leakage of the microcapsules constituents into the bulk solution occurred, as evidenced by precipitation of the microcapsules mixture (after three days) and subjecting the solution to glucose and Amplex Red. Therefore, the loaded microcapsules M_1, M_2 mixture retained their intact compositions and activities after a time-interval of three days. Resuspension of the precipitated M_1/ M_2 mixture revealed upon addition of glucose and Amplex Red, the initial biocatalytic activity shown in Figure 6 (B), curve (i).

Following the Dimerization of Coumarin-modified GOx-loaded Microcapsules

As the coumarin-modified GOx-loaded microcapsules include on their hydrogel boundaries the complementary p and q tethers originating from the HCR process, *cf.* Figure 5, the dynamic self-dimerization of the microcapsules is anticipated. Figure S18 (A) exemplifies confocal fluorescence images of the monomer/dimers present in the mixture. By analyzing ten different fluorescence domains associated with three different samples of the microcapsules (total 30 field frames), the respective contents of the monomer/dimer-constituents were evaluated and these are presented in Figure S18 (B). The mixture includes *ca.* 35% of dimers and 52% monomers and *ca.* 13% of non-clearly defined structures.

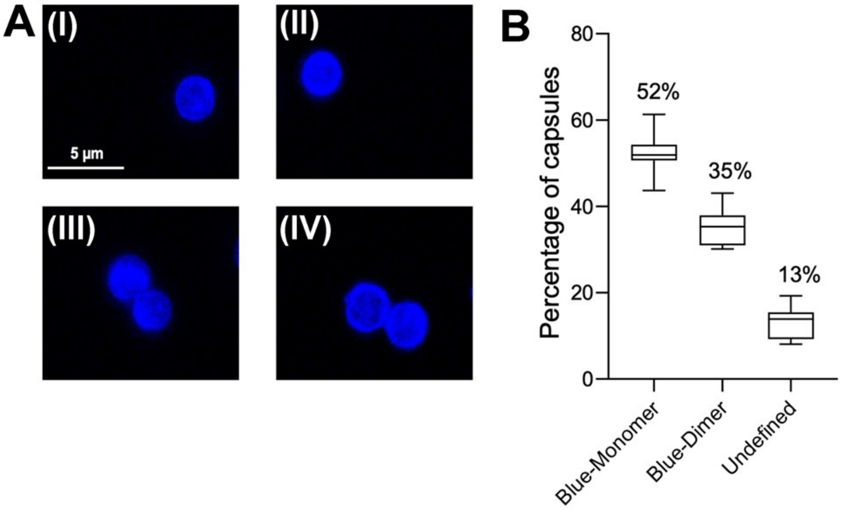

Figure S18 (A) Examples of confocal microscopy images corresponding to Coumarin-labeled GOx-loaded microcapsules. Panel I and II-Monomer microcapsules. Panel III and IV-Dimer microcapsules. (B) Statistical analysis confocal fluorescence microscopy fields of the population of different monomer/dimer microcapsules assemblies (total 30 fields). (E_x=405 nm, E_m=430-470 nm) Error bars derived from N = 3 experiments.

Following the Dimerization of Cy5-labeled T_1/T_2-loaded Microcapsules

As the Cy5-labeled T_1/T_2-loaded microcapsules include on their hydrogel boundaries the complementary p and q tethers originating from the HCR process, *cf.* Figure 5, the dynamic self-dimerization of the microcapsules is anticipated. Figure S19 (A) exemplifies confocal fluorescence images of the monomer/dimers present in the mixture. By analyzing ten different fluorescence domains associated with three different samples of the microcapsules (total 30 field frames), the respective contents of the monomer/dimer-constituents were evaluated and these are presented in Figure S19 (B). The mixture includes *ca.* 48% of dimers and 32% monomers and *ca.* 13% of non-clearly defined structures.

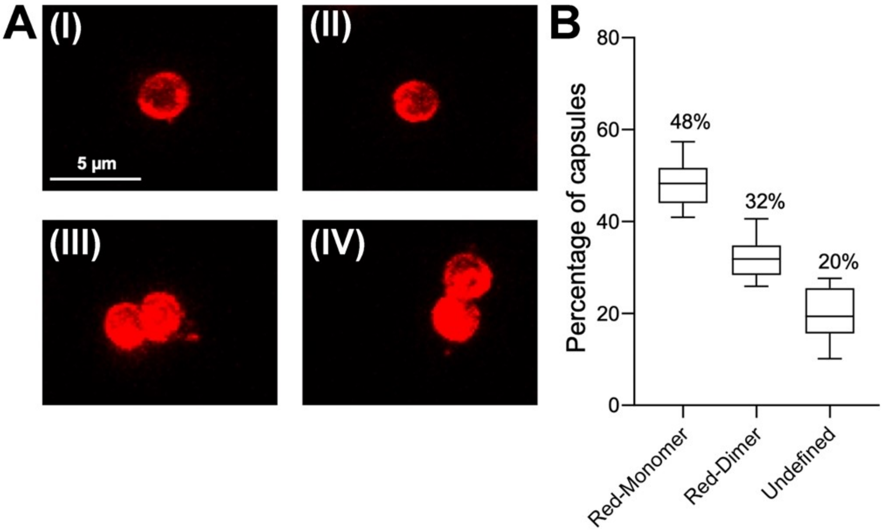

Figure S19 (A) Examples of confocal microscopy images corresponding to Cy5-T_1/T_2-loaded microcapsules. Panel I and II-Monomer microcapsules. Panel III and IV-Dimer microcapsules. (B) Statistical analysis confocal fluorescence microscopy fields of the population of different monomer/dimer microcapsules assemblies (total 30 fields). (E_x=640 nm, E_m=650-700 nm) Error bars derived from N = 3 experiments.

FACS Analysis of the Microcapsule Mixture Consisting of the Coumarin-Labeled GOx-Loaded Capsules and the Cy5-Labeled T_1/T_2-Loaded Microcapsules

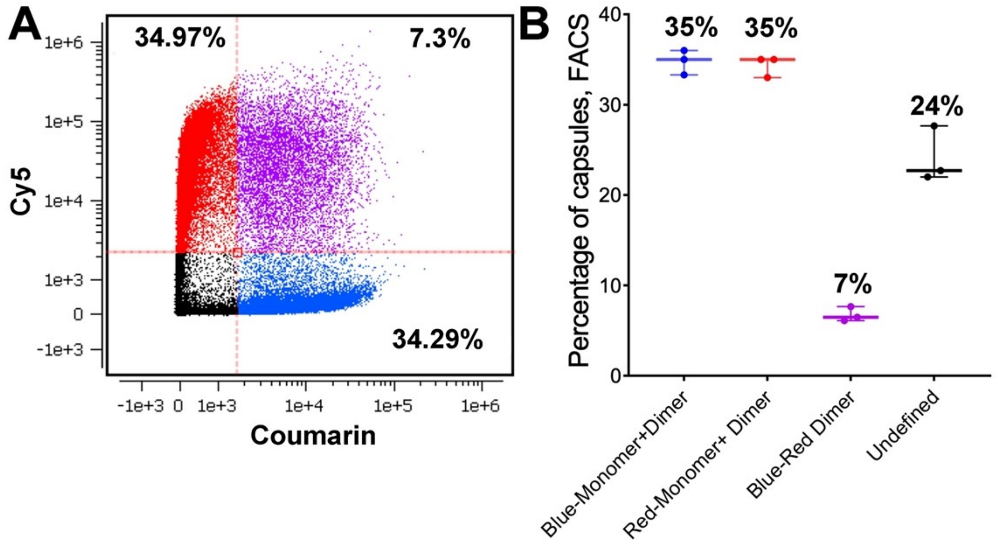

Figure S20 FACS analysis of the mixture consisting of coumarin-labeled GOx-loaded microcapsules and the Cy5-labeled T_1/T_2-loaded microcapsules. (A) Microcapsules distribution depending on the fluorescence intensity of the labeled fluorophores; the upper right quartet (Violet dots) representing the dimer composed of the two fluorophores capsules, the upper left quarter (red dots) representing the monomers and dimers of the red fluorescence of the Cy5-labeled T_1/T_2- loaded capsules, the lower right quarter (blue dots) corresponding the monomers and dimers of the red fluorescence of the coumarin-labeled GOx-loaded microcapsules, and the lower left quarter corresponding the undefined structures with no fluorescence. (B) Graph representing the percentage of each color combinations of the fluorescent capsules from the sample mixture. Results are presented as box and whiskers plots and include all data points measured in three different experiments. Error bars derived from *N* = 3 experiments.

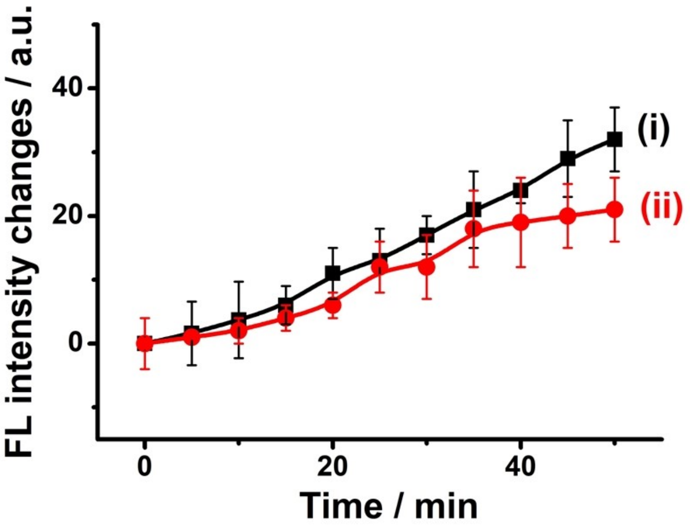

Figure S21 Time-dependent fluorescence intensity of Resorufin generated by M_1/M_2 microcapsule mixture, GOx-loaded microcapsules, and hemin/G-quadruplex DNAzyme-bridged tetrahedra loaded microcapsules as a result of: curve (i) Upon separation of the dimers using p' and q' fuel strands, (state II depicted in Figure 6 (A)). Curve (ii) allowing the dimer-containing microcapsule mixture to interact for a time-interval of ten days and treatment of the mixture after this time-interval with p', q' to induce separation of dimer microcapsules. In all measurements, K^+-ions, 50 mM, glucose 30 mM, hemin, 0.167 μM, and Amplex Red, 0.083 mM were subjected to the system. Error bars derived from $N = 3$ experiments.
